# BIK polymorphism and proteasome regulation unveil host risk factor for severe influenza

**DOI:** 10.1073/pnas.2424367122

**Published:** 2025-07-08

**Authors:** Sourabh Soni, Soner Yildiz, Emma Kaitlynn Allen, Hans Petersen, Mark Peeples, Sara El Zahed, Lorena Rosas, Vandana Anang, Laura Antonescu, Richard Seonghun Nho, Ana Lucia Mora, Jeffrey Craig Horowitz, Mauricio Rojas, Rafael Andrés Medina, Paul Glyndwr Thomas, Adolfo García-Sastre, Yohannes Tesfaigzi, Yohannes Afework Mebratu

**Affiliations:** ^a^Department of Internal Medicine, Division of Pulmonary, Critical Care, and Sleep Medicine, Davis Heart and Lung Research Institute, College of Medicine, The Ohio State University Wexner Medical Center, Columbus, OH 43210; ^b^Department of Microbiology, Icahn School of Medicine at Mount Sinai, Mount Sinai, NY 10029; ^c^Global Health and Emerging Pathogens Institute, Icahn School of Medicine at Mount Sinai, Mount Sinai, NY 10029; ^d^Department of Immunology, St. Jude Children’s Research Hospital, Memphis, TN 38105; ^e^Chronic Obstructive Pulmonary Disease Program, Lovelace Respiratory Research Institute, Albuquerque, NM 87108; ^f^Center for Vaccines and Immunity, Abigail Wexner Research Institute at Nationwide Children’s Hospital, Columbus, OH 43205; ^g^Pathology Advanced Translational Research Unit, Department of Pathology and Laboratory Medicine Emory Vaccine Center, Emory University School of Medicine, Atlanta, GA 30322; ^h^Department of Medicine, Division of Infectious Diseases, Icahn School of Medicine at Mount Sinai, Mount Sinai, NY 10029; ^i^The Tisch Cancer Institute, Icahn School of Medicine at Mount Sinai, Mount Sinai, NY 10029; ^j^Department of Pathology, Molecular and Cell-Based Medicine, Icahn School of Medicine at Mount Sinai, Mount Sinai, NY 10029; ^k^The Icahn Genomics Institute, Icahn School of Medicine at Mount Sinai, Mount Sinai, NY 10029; ^l^Division of Pulmonary and Critical Care Medicine, Brigham and Women’s Hospital, Harvard Medical School, Boston, MA 02115

**Keywords:** Influenza A virus, BIK, Proteasome, SNP, Beta 5

## Abstract

Influenza A viruses (IAVs) pose a significant public health threat, with host factors playing a crucial role in disease severity. We investigated the role of Bcl-2-interacting killer (BIK) in IAV infection using cellular and mouse models, and influenza-infected human cohort. In airway epithelial cells (AECs), BIK deficiency impaired viral replication, while BIK restoration enhanced it. Conversely, airway-specific BIK overexpression in mice increased viral load, inflammation, and mortality, whereas BIK suppression conferred protection. Critically, a genetic variation (rs738276) in the *BIK* gene, influencing BIK expression, correlates with altered viral replication in air–liquid interface differentiated primary normal human bronchial epithelial cells and influenza severity in humans. Mechanistically, we demonstrate that IAV nucleoprotein (NP) suppresses β5, a subunit of the proteasome, leading to increased BIK levels and enhanced viral replication. Conversely, β5 treatment dampened BIK levels and protected mice from IAV-induced morbidity and mortality. Furthermore, BIK interacts with NP, disrupting the Bcl-2/NP interaction and promoting viral replication. Our findings uncover an IAV–BIK–β5 axis that governs viral replication, suggesting that targeting BIK or β5 may offer therapeutic strategies against influenza.

## Significance

We identify Bcl-2-interacting killer (BIK) as a critical host factor for influenza A virus (IAV) replication, revealing an IAV–BIK–β5 axis. This axis demonstrates how the viral nucleoprotein manipulates host cell machinery to promote viral replication in the airways. Furthermore, we show that genetic variation in BIK affects viral replication in the airway epithelial cells and correlates with human influenza severity. These findings provide critical insights into the host–virus interaction and suggest potential therapeutic targets for influenza.

Influenza remains a pressing global health concern, causing substantial morbidity and mortality each year. With an estimated 1 billion cases and up to 650,000 associated deaths annually, its impact is profound (1). The economic toll of seasonal influenza in the United States is staggering, exceeding $87.1 billion annually, including direct medical costs surpassing $10.4 billion (2, 3). Despite the availability of antiviral drugs (4, 5), the emergence of resistance (6–9), and the zoonotic potential of avian strains (10, 11), underscores the need for novel therapeutic strategies. To address this challenge, a shift in focus from current drug targets to the development of universal treatments or vaccines effective against all viral strains is crucial. Since viruses rely on host cellular machinery for replication and can subvert normal host functions (12), uncovering host–virus interactions is essential to understanding viral replication mechanisms, evasion of host defenses (13), and identification of key host factors (14).

Host cell death regulation is intrinsically linked to influenza A virus (IAV) pathogenesis, with Bcl-2 family proteins playing a complex role in modulating apoptosis (15, 16). While prosurvival Bcl-2 generally inhibits viral replication (17, 18), proapoptotic members, including Bcl-2-interacting killer (BIK) (19), Bax (20), and Bad (21), have been implicated in promoting it. The ubiquitin proteasome system (UPS) also plays a pivotal role in viral pathogenesis, exhibiting both pro- and antiviral effects (22–24). Viruses manipulate the UPS to enhance viral protein function or facilitate infection by regulating viral or host protein stability (23, 25–28). Our recent work has suggested a role for BIK in IAV replication (19, 29), and evidence indicates that BIK levels can be modulated by the proteasome (30–33). However, the precise mechanisms by which IAVs exploit BIK to promote replication within airway epithelial cells (AECs) remain incompletely understood.

Here, we investigate how IAVs regulate BIK levels to enhance viral replication in AECs and contribute to disease progression in the lungs. We demonstrate that BIK deficiency impairs viral replication in AECs, whereas BIK restoration enhances it. Conversely, airway-specific BIK overexpression in mice increases lung viral load, inflammation, and mortality, while BIK suppression confers protection. Building upon our previous finding that the *BIK* promoter single nucleotide polymorphism (SNP) rs738276 influences BIK expression (34), we now demonstrate a direct correlation between this genotype and enhanced viral replication. Specifically, air–liquid interface (ALI)-differentiated primary normal human bronchial epithelial cells (NHBEs) from individuals with the rs738276 AA genotype, known to exhibit elevated BIK expression, displayed significantly increased IAV replication, consistent with its association with severe influenza. Mechanistically, IAV nucleoprotein (NP) suppresses the proteasome’s catalytic subunit β5, inhibiting BIK degradation. IAV-stabilized BIK interacts with NP, disrupting the Bcl-2/NP interaction and ultimately enhancing viral replication. These findings uncover an IAV–BIK–β5 axis governing viral replication, suggesting BIK and β5 as potential therapeutic targets for influenza susceptibility and severity. Furthermore, screening for rs738276 could identify individuals at heightened risk for severe influenza outcomes, opening possibilities for targeted prevention and treatment.

## Results

### BIK Deficiency Suppresses IAV Replication in the AECs.

**Fig. 1.**
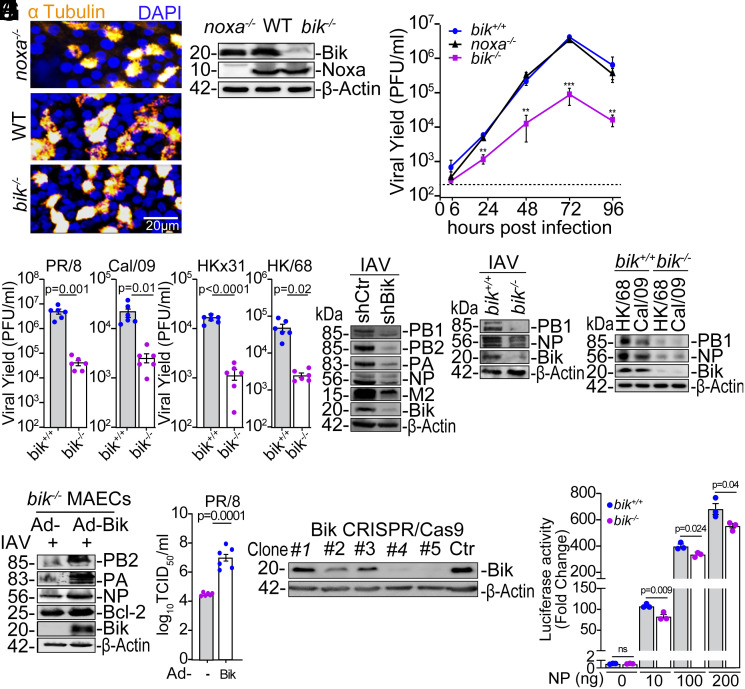
BIK is essential for IAVs replication. (*A*) Cells from ALI-differentiated uninfected WT (*bik^+/+^*), *bik^−/−^*, and *noxa^−/−^* primary MAECs, shown by immunofluorescence (*Left* panel) were subjected to Western blot (*Right* panel). α-tubulin was used to stain differentiated cells. (*B*) ALI-differentiated primary MAECs were infected with 0.1 MOI PR/8 for the indicated time points. Viral titers were analyzed in the apical washes using plaque assay. n = 6/group. One-way ANOVA. (*C*) ALI-differentiated primary *bik^+/+^* and *bik^−/−^* MAECs were infected with 0.1 MOI of the indicated IAVs. Viral titers were analyzed 72 hpi by plaque assay. n = 6/group. Two-tailed *t* test. (*D*) Human AECs stably expressing shCtr or shBIK, or *bik^+/+^* and *bik^−/−^* MAECs were infected with (*E*) 1 MOI PR/8 or (*F*) 1 MOI HK/68 or Cal/09. Protein lysates were analyzed 24 hpi. (*G* and *H*) Primary *bik^−/−^* MAECs differentiated in ALI were infected with 0.1 MOI PR/8 and treated with 50 MOI of an empty adenoviral vector (Ad-) or Ad-BIK: (*G*) Protein lysates were analyzed 72 hpi. (*H*) Viral titers were analyzed in the apical washes 72 hpi by TCID_50_. n = 6/group. Two-tailed *t* test. (*I*) HEK293T cells were infected with lentiviral vector for BIK CRISPR and Cas9. *BIK* knockout clones were identified through selection using puromycin and BIK expression level was compared between clones by Western blot. Clone #5 was used for the subsequent studies. (*J*) Parent and CRISPR/Cas9 *BIK* knockout HEK293T cells were cotransfected with PA, PB1, PB2, and NP plasmids from PR/8 on a pCAGGS backbone, and firefly and renilla luciferase plasmids. The firefly luciferase/renilla ratio is used to calculate the luciferase (polymerase) activity. n = 3/group. Two-tailed *t* test. Error bars: Mean ± SEM. **P < 0.01; ***P < 0.001. Western blotting experiments were performed in triplicate, and representative images are presented.

Previous studies have implicated Bcl-2 family proteins in IAV replication (17, 19–21, 29, 35), but the precise mechanisms remain unclear. To investigate the role of BIK, a proapoptotic BH3-only protein, we examined IAV replication in primary mouse AECs (MAECs). We differentiated MAECs from wild-type (WT), *bik*-deficient (*bik^−/−^*) mice, and mice lacking another proapoptotic BH3-only protein of the Bcl-2 family (*noxa^−/−^*) (36) (Fig. 1*A*) and infected them with 0.1 MOI A/PR/8/34 (H1N1) (PR/8) virus. Viral titers in the apical washes were measured 6 to 96 h post infection (hpi). Viral titer was significantly reduced in *bik^−/−^* compared with the WT (*bik^+/+^*) and *noxa^−/−^* cells 24 to 96 hpi with the peak differences observed at 72 hpi (Fig. 1*B*). Noxa deficient cells showed no significant difference in viral replication compared to WT, indicating that the observed reduction in virus replication in *bik^−/−^* cells is specific to BIK deficiency and not a general effect of BH3-only protein loss. To investigate whether BIK’s proviral role extends to other IAV strains, we infected *bik^+/+^* and *bik^−/−^* AECs with 0.1 MOI PR/8, A/Cal/07/09 (H1N1) (Cal/09), and two viruses possessing the HA and NA surface glycoproteins derived from A/Hong Kong/1/1968 (H3N2): A/HKx31 and A/HK/2/68 (HK/68). Viral titers in the apical washes at 72 hpi were diminished by 10- to 100-fold in *bik^−/−^* compared with *bik^+/+^* cells across all the viruses tested (Fig. 1*C*). Consistent with reduced viral replication, viral protein levels were reduced in *BIK* knockdown human AECs (HAECs) (Fig. 1*D*) and *bik^−/−^* MAECs when infected with PR/8 (Fig. 1*E*) or HK/68 and Cal/09 (Fig. 1*F*). To confirm that the reduced viral replication in *bik^−/−^* cells was specifically due to BIK deficiency, we ectopically expressed BIK in *bik^−/−^* MAECs using an adenoviral vector (Ad-BIK) and assessed viral replication. Compared to cells transduced with an empty vector (EV) (Ad-), Ad-BIK expression restored viral protein levels (Fig. 1*G*) and rescued viral replication (Fig. 1*H*). Next, we infected *bik^+/+^* and *bik^−/−^* cells with 0.1 MOI PR/8 and analyzed the viral NP positivity at 4 to 72 hpi. Immunostaining revealed that while there were some NP-positive *bik^−/−^* cells as early as 4 hpi; the NP-positivity in the *bik^+/+^* cells continued to increase from 4 to 72 hpi. In contrast, there was very little increase in percentage of NP positive cells in the *bik^−/−^* cells (*SI Appendix*, Fig. S1 *A* and *B*). Furthermore, using luciferase-based minigenome reporter assay with PR/8, we found that IAV polymerase activity was significantly reduced in CRISPR/Cas9 *bik^−/−^* HEK293T cells compared to *bik^+/+^* cells (Fig. 1 *I* and *J* and *SI Appendix*, Fig. S1*C*). Collectively, these data suggest that *bik^−/−^* cells are not resistant to initial infection, but rather BIK serves as an important host factor for efficient viral replication.

### Airway-Specific BIK Overexpression Augments IAV Replication, Lung Inflammation, and Mortality in Mice.

**Fig. 2.**
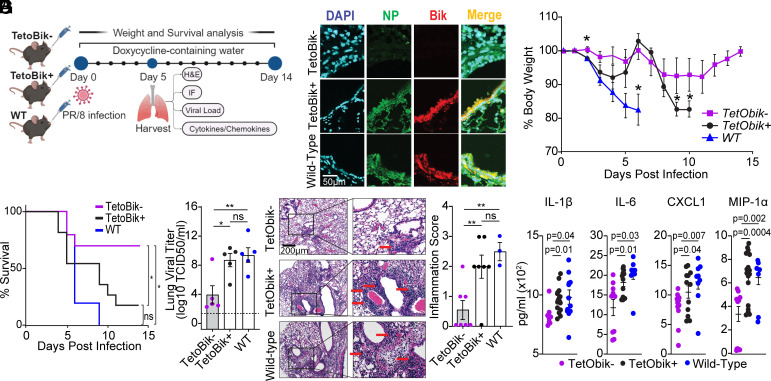
Airway-specific BIK overexpression enhances IAV-induced morbidity and mortality in mice. (*A*) Eight weeks old WT, CCSP-IndBik transgenic mice in *bik^−/−^* background and their littermates were infected with 250 pfu PR/8 intranasally and kept on 400 mg/L doxycycline containing water ad libitum. Figure made with Biorender.com. (*B*) BIK and viral NP protein expression levels were analyzed in the lungs of mice by immunostaining on 5 dpi. Mice were monitored for (*C*) changes in body weight (one-way ANOVA) and (*D*) survival (Log-rank (Mantel-Cox) test) for a period of 14 d. n = 10/group (5 males and 5 females). (*E*) Lung viral load was analyzed using the median tissue culture infectious dose (TCID_50_) 5 dpi. The lowest detection limit for the TCID_50_ is 10^2^. n = 5/group; One-way ANOVA. (*F*) Microscopic evaluation of lung sections stained with H&E for histopathological analysis at 5 dpi. Red arrows indicate inflammatory cell infiltration in the alveoli and septa. Inflammation score was calculated in a blind manner by a certified pathologist. One-way ANOVA; n = 3 to 7/group. (*G*) Proinflammatory cytokine and chemokine levels in the lung homogenates analyzed at 5 dpi using Luminex multiplex assay. ANOVA; n = 8 to 16/group. Error bars: Mean ± SEM; *P < 0.05, **P < 0.01, ns = not significant.

Our previous study showed that, compared with the *bik^+/+^* (WT), *bik^−/−^* mice exhibit reduced lung inflammation, lower viral loads, and improved survival following lethal IAV infection (19). To further explore BIK’s role in IAV-induced morbidity and mortality, we used WT, doxycycline (dox)-inducible *BIK*-transgenic (CCSP-rtTA+/TetObik+) and littermate control (CCSP-rtTA+/TetObik−) mice on *bik^−/−^* background (*SI Appendix*, Fig. S2*A*). We infected these mice with a lethal dose (250 pfu) of PR/8 intranasally and kept them on dox-containing water ad libitum to induce the transgene (Fig. 2*A*). We observed increased expression and colocalization of BIK and NP in the airways of WT and TetObik+ mice (Fig. 2*B*). TetObik+ mice exhibited greater weight loss (Fig. 2*C*) and reduced survival (Fig. 2*D*) compared to TetObik− mice. While all WT mice succumbed to infection by day 9, and 80% of TetObik+ mice died by day 11 from infection, 60% of TetObik− mice survived the challenge. Stratification of weight loss and survival data by sex revealed that both male and female TetObik+ mice exhibited significantly greater weight loss (*SI Appendix*, Fig. S2*B*) and reduced survival (*SI Appendix*, Fig. S2*C*) compared to TetObik− mice, indicating that these effects were independent of sex. Consistent with these findings, compared with the TetObik−, the WT and TetObik+ mice showed higher viral loads (Fig. 2*E*) and more severe lung inflammation (Fig. 2*F*). Analysis of inflammatory mediators in the lung homogenates using a Luminex multiplex assay revealed significantly elevated levels of proinflammatory mediators IL-1β, IL-6, CXCL1, and MIP-1α in WT and TetObik+ mice compared to TetObik− mice (Fig. 2*G*). The observed differences in viral loads and lung inflammation are in agreement with morbidity and mortality data. Collectively, these results demonstrate that airway-specific overexpression of BIK significantly enhances IAV replication in the lungs, leading to exacerbated lung damage and increased mortality.

### Airway-Specific BIK Silencing Mitigates IAV-Induced Mortality in Mice.

To assess the therapeutic potential of BIK silencing in the airways, we instilled WT C57BL/6 mice with retroviral vectors expressing either control shRNA (shCtr) or shRNA targeting BIK (shBIK) intranasally 2 d prior to infection with a lethal dose of PR/8 (*SI Appendix*, Fig. S3*A*). Analysis of lung tissues 1 d post-infection (dpi) confirmed effective *BIK* silencing in the airways of shBIK-treated mice (*SI Appendix*, Fig. S3*B*). While mice treated with shCtr showed a drastic decrease in body weight, the shBIK-treated mice lost less weight and recovered by day 14 (*SI Appendix*, Fig. S3*C*). Notably, all shCtr-treated mice succumbed to infection by 5 dpi, whereas 80% of the shBIK-treated mice survived (*SI Appendix*, Fig. S3*D*), suggesting that reducing BIK levels in the airways significantly decreases susceptibility to IAV-induced morbidity and mortality. To evaluate viral load and lung pathology, lungs were collected at 5 dpi. Compared to shCtr-treated mice, shBIK-treated mice showed diminished NP-positivity (*SI Appendix*, Fig. S3*E*), a significant reduction in viral load (*SI Appendix*, Fig. S3*F*), and decreased lung inflammation (*SI Appendix*, Fig. S3*G*). Sex-stratified analysis revealed that both male and female mice treated with shBIK exhibited significantly reduced weight loss (*SI Appendix*, Fig. S3*C*), increased survival (*SI Appendix*, Fig. S3*D*), decreased viral load (*SI Appendix*, Fig. S3*F*), and diminished inflammation (*SI Appendix*, Fig. S3*G*) compared to shCtr-treated mice, demonstrating that *BIK* knockdown conferred protection regardless of sex. These findings indicate that merely reducing the BIK levels in the airways is sufficient to decrease IAV replication, mitigate IAV-induced lung inflammation, and protect mice from lethal IAV infection.

### A Single-Nucleotide Polymorphism (SNP) in the *BIK* Gene Affects BIK Level and IAV Replication.

**Fig. 3.**
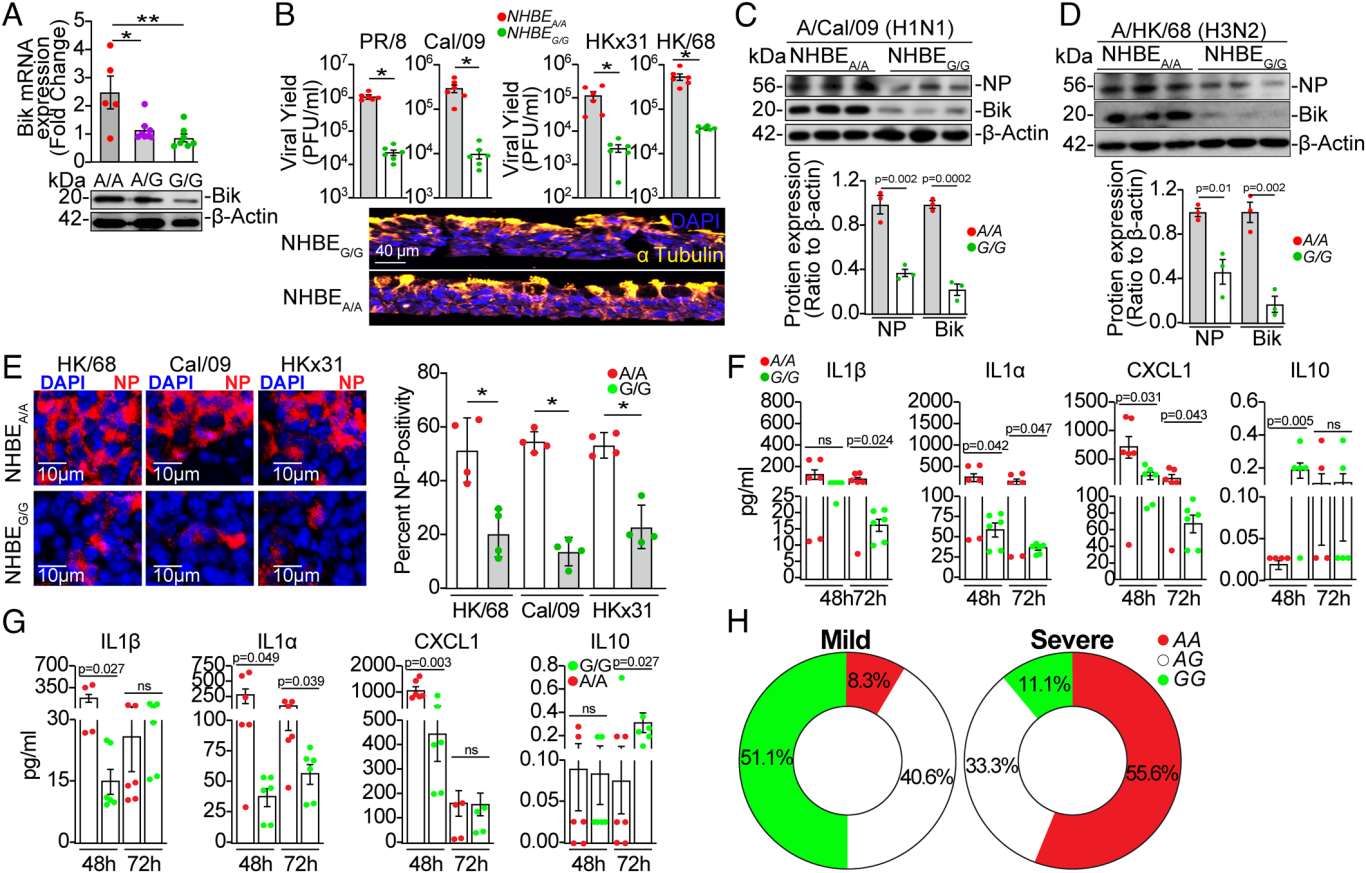
*BIK* SNP affects its expression and IAV replication. Primary NHBEs from people with AA, AG, or GG variant of *BIK* SNP (rs738276) were differentiated in ALI culture. (*A*) *BIK* mRNA and protein expression levels were analyzed by qRT-PCR and Western blot, respectively. (*B*) Differentiated primary NHBEs cultures from individuals with AA or GG variants of the *BIK* SNP were infected with 0.1 MOI PR/8, Cal/09, HKx31, or HK/68. Apical washes were analyzed for virus titers using plaque assay 72 hpi. The tissue section below the graph shows staining of the differentiated cultures for α-tubulin (marker for ciliated cells) and DAPI (nuclear staining). Two-tailed *t* test; n = 6/group. (*C* and *D*) Protein lysates from differentiated cultures infected with (*C*) H1N1 or (*D*) H3N2 subtypes were analyzed for viral NP and BIK levels 24 hpi. Relative protein expression was calculated by densitometric analysis (ratio to β-actin) and normalized to AA. n = 3/group. (*E*) Differentiated cultures from the indicated *BIK* SNP were infected with the indicated strains of IAV and immunostained for viral NP and analyzed by confocal microscopy 24 h later. Percent NP-positivity was analyzed using ImageJ software. Two-tailed *t* test; n = 4/group. (*F* and *G*) Inflammatory cytokine and chemokine levels in the supernatants of NHBEs infected with (*F*) Cal/09 or (*G*) HK/68 were analyzed using Luminex multiplex assay. *t* test; n = 6/group. (*H*) Genotype frequency distribution of rs738276 in FLU09 cohort of naturally acquired influenza infection (n = 105) in mild influenza illness phenotypes (*Left*) compared with severe influenza illness phenotypes (*Right*). Western blotting experiments were performed in triplicate, and representative images are presented. Error bars: Mean ± SEM. *P < 0.05, **P < 0.01.

Our previous study identified the *BIK* promoter SNP rs738276 and demonstrated its impact on BIK expression (34). Specifically, we showed that individuals with the AA genotype exhibited higher *BIK* mRNA and protein levels compared to those with the GG genotype, while AG heterozygotes showed intermediate levels (34). Here, we investigated the functional consequences of this SNP on viral replication. Using well-differentiated primary NHBEs (Fig. 3*A*) and peripheral blood mononuclear cells (PBMCs) (*SI Appendix*, Fig. S4), we now show that the AA variant also exhibits higher BIK expression compared to the GG variant in these cell types. We report that in NHBE cultures infected with PR/8, Cal/09, HKx31, or HK/68; cells with the AA variant (NHBE_A/A_) showed significantly higher viral loads compared with the GG variant (NHBE_G/G_) (Fig. 3*B*). Similarly, NP expression levels were also elevated in NHBE_A/A_ cells across these infections (Fig. 3 *C*–*E*). These results are in agreement with elevated BIK protein levels in NHBE_A/A_, compared to NHBE_G/G_ cultures that produce low BIK protein levels in response to influenza infections.

Analysis of inflammatory mediators in NHBE culture supernatants (Luminex multiplex assay) revealed that IL1β, IL1α, and CXCL1 were significantly elevated in NHBE_A/A_ cultures compared to NHBE_G/G_ cultures following infection with Cal/09 and HK/68 (Fig. 3 *F* and *G*). Conversely, the anti-inflammatory cytokine IL10 was reduced in NHBE_A/A_ cultures after infection with both viruses (Fig. 3 *F* and *G*).

### The AA Variant of the *BIK* SNP Is Associated with Influenza Disease Severity in Humans.

Since viral replication (Fig. 3*B*) and protein levels (Fig. 3 *C*–*E*) were increased in NHBE_A/A_ compared with NHBE_G/G_, we investigated whether the *BIK* SNP that affects its expression is associated with influenza disease severity. We used the FLU09 cohort, a longitudinal study of naturally acquired influenza infection, where illness severity was defined using clinical and symptom data (37). This cohort, recruited at St. Jude Children’s Hospital and the University of Tennessee, is predominantly African American (80%) with 20% Caucasian participants (n = 105) (Table 1). Symptomatic data, including systemic, upper respiratory, lower respiratory, and gastrointestinal symptoms, were collected daily and ranked using a visual analog scale. Study participants were stratified into mild and severe illness groups based on their total peak symptom score (37, 38). Analysis of the genotype clustering data using the Quant Studio Software (Applied Biosystems) showed that 13.3%, 40%, and 47.6% carried AA, AG, and GG genotypes, respectively. Chi-squared comparison of AA vs. AG/GG showed that the AA allele of *BIK* SNP is significantly associated with severe influenza as defined by the total symptom score (*P* = 0.0002). Recessive model analysis revealed that the AA allele was significantly associated with increased influenza disease severity defined by lower respiratory symptoms (*P* = 0.0001) and total symptom score (*P* = 0.0001) (Table 1). Age, sex, race, and hospital visits were not significantly different among people with different genotypes. Logistic regression analysis adjusted for age, sex, and race showed significant association between the GG allele and low total symptom score compared to the AA/AG alleles combined (*P* = 0.0354). In patients with severe symptoms defined by total symptom score, the AA, AG, and GG variants accounted for 55.6%, 33.3%, and 11.1%, respectively (Fig. 3*H*). Further, logistic regression analysis of the data for total symptom score (high vs. low) adjusted for age, sex, and race showed significant risk for the AA allele compared with AG or GG [OR = 65.2 (CI: 3.17 to 1338), *P* = 0.0067]. Linear regression, adjusted for age, sex, and race, also showed a significant association between the AA allele and increased influenza severity compared to AG or GG variants [PE = 284.7 (SE = 62.8), *P* < 0.0001]. The association of the promoter *BIK* SNP, shown to alter BIK expression levels, with severity of influenza-caused disease symptoms in humans supports the effect of BIK on viral replication in the AECs.

**Table 1.** Analysis of association between *BIK* SNP and influenza severity (AA vs. GG/AG)

Characteristic	Total (N = 105) N or mean	*BIK* AA (N = 14) N or mean	*BIK* AG/GG (N = 91) N or mean	*P* value
Age (SD)	16.1 (15.0)	18.8 (17.8)	15.6 (14.6)	0.467
Male (%)	46 (43.8)	5 (35.7)	41 (45.1)	0.512
African American (%)	84 (80.0)	11 (78.6)	73 (80.2)	0.886
Non-Hispanic White (%)	21(20.0)	3 (21.4)	18 (19.8)	0.886
Hospital (Yes vs. No) (%)	15 (18.1)	2 (15.4)	13 (18.6)	0.784
Hospital or ER vs. Not Hospital (%)	32 (38.6)	4 (30.8)	28 (40.0)	0.530
Lower respiratory symptom (Yes vs. No) (%)	27 (37.5)	11 (84.6)	16 (27.1)	0.0001
Total symptom score (High vs. low) (%)	10 (13.9)	6 (46.2)	4 (6.8)	0.0001
Total symptom score (Continuous) (SD)	269 (263.5)	520.8 (401)	213.4 (185)	<0.0001

**Fig. 4.**
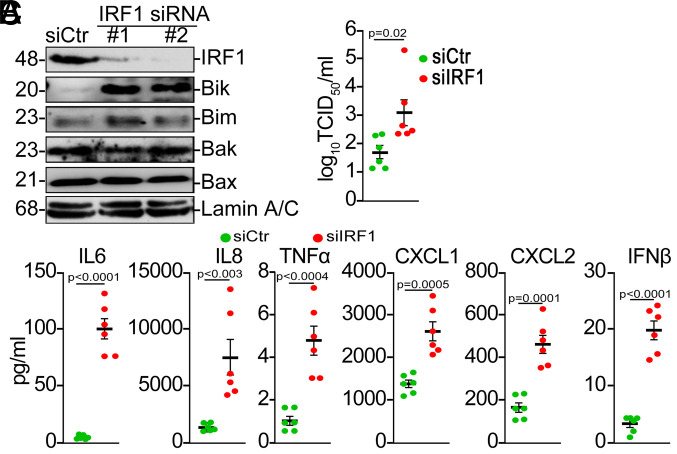
IRF1 silencing upregulates BIK, promoting viral replication and inflammation. NHBEs with the GG variant of the *BIK* SNP (NHBE_G/G_) were transfected with siControl or siIRF1 followed by infection with 0.1 MOI Cal/09 48 h posttransfection. (*A*) Protein lysates were analyzed for BIK, Bim, Bax, Bak, and IRF1 levels. (*B*) Viral titers and (*C*) inflammatory cytokine and chemokine levels in cell supernatants were compared at 72 hpi using TCID_50_ assay and Luminex multiplex assay, respectively. *t* test; n = 6/group. Error bars: Mean ± SEM.

We recently discovered that the sequence encompassing rs738276 shares high homology with the consensus binding site for interferon regulatory factor-1 (IRF-1). IRF-1 preferably binds to the *BIK* intronic region with the G allele, suppressing BIK expression (34). This suggests that IRF-1 negatively regulates BIK expression. Given IRF-1’s role in regulating BIK expression, we hypothesized that suppressing IRF-1 in NHBE_G/G_ cells would increase BIK expression, leading to enhanced viral replication and subsequent increases in proinflammatory mediators. To test this, we used siRNA to silence IRF-1 in NHBE_G/G_ cells. IRF-1 silencing effectively increased BIK expression without affecting the expression of other proapoptotic proteins such as Bim, Bak, Bax, and Bad (Fig. 4*A* and *SI Appendix*, Fig. S5), suggesting specific effect on BIK. Furthermore, IRF1 silencing enhanced viral replication (Fig. 4*B*) and significantly upregulated proinflammatory cytokines and chemokines (Fig. 4*C*). These findings suggest that the *BIK* SNP rs738276 plays a significant role in regulating BIK expression, which in turn impacts IAV replication and the inflammatory response.

### IAV NP Inhibits 20S Proteasome Activity, Stabilizing BIK Protein.

**Fig. 5.**
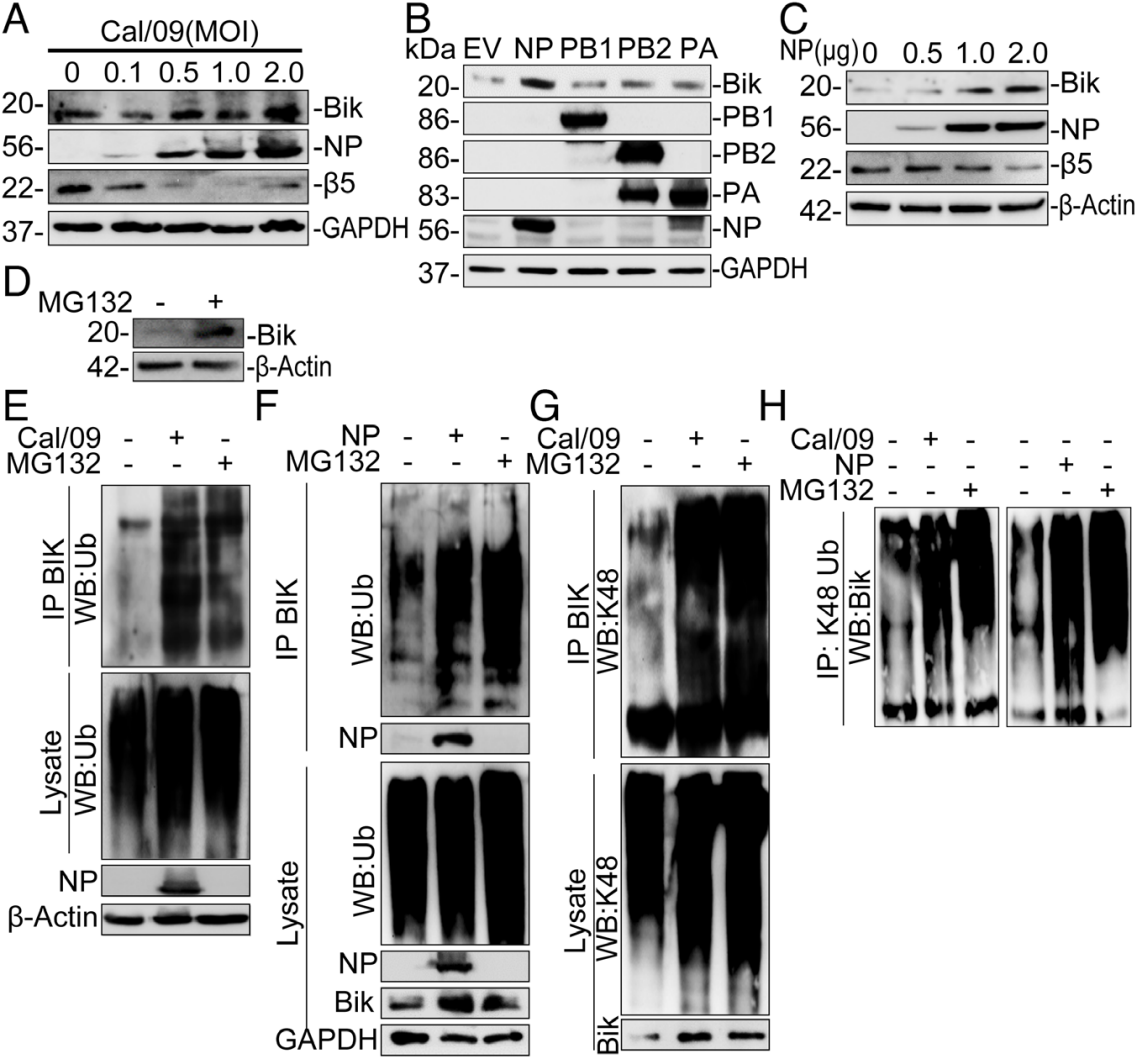
IAV-NP inhibits proteasomal degradation of BIK. (*A*) HAECs were infected with 0 to 2 MOI Cal/09 and protein lysates analyzed for BIK, NP, and β5 expression. (*B*) HEK293T cells were transfected with 1 µg EV or the indicated viral protein constructs from Cal/09. (*C*) HEK293T cells were transfected with 0-2 µg Cal/09 NP plasmid. Protein lysates were analyzed for BIK, β5, and viral protein expression levels. (*D*) HEK293T cells were treated with vehicle or 20 µM MG132 for 6 h. Protein lysates were analyzed for BIK expression level. (*E* and *F*) HEK293T cells were (*E*) infected with mock (infection media) or 0.1 MOI Cal/09 or (*F*) transfected with 1.0 µg EV or Cal/09 NP-expressing plasmid. Forty-eight hours later, the cells were treated with 20 µM MG132 for 6 h. Protein lysates were immunoprecipitated with anti-BIK antibody and analyzed for BIK ubiquitination using anti-ubiquitin antibody. (*G* and *H*) HEK293T cells were (*G*) infected with mock or 0.1 MOI Cal/09 or (*H*) transfected with EV or Cal/09 NP-expressing plasmid. Forty-eight hours later, protein lysates were immunoprecipitated with (*G*) anti-BIK or (*H*) anti-K48 ubiquitin antibodies and were analyzed for (*G*) K48 or (*H*) BIK. Western blotting experiments were performed in triplicate, and representative images are presented.

To explore the mechanism of how BIK promotes viral replication, we first tested whether IAV infection regulates BIK expression. While IAV infections did not affect the mRNA levels of Bcl-2 family genes, including *BIK* [except for BAD, as previously reported (21)] in human AECs (*SI Appendix*, Fig. S6*A*), it did induce BIK protein expression in a dose-dependent manner (Fig. 5*A*). To identify the specific viral protein responsible, we transfected HEK293T cells with EV or plasmids expressing IAV-NP, PB1, PB2, or PA proteins derived from Cal/09 strain and analyzed the BIK level. We found that NP, but not other components of the viral ribonucleoprotein (vRNP), increased BIK protein levels (Fig. 5*B*) in a dose-dependent manner (Fig. 5*C*). Similarly, NP expression induced BIK protein in A549 cells (*SI Appendix*, Fig. S6*B*).

**Fig. 6.**
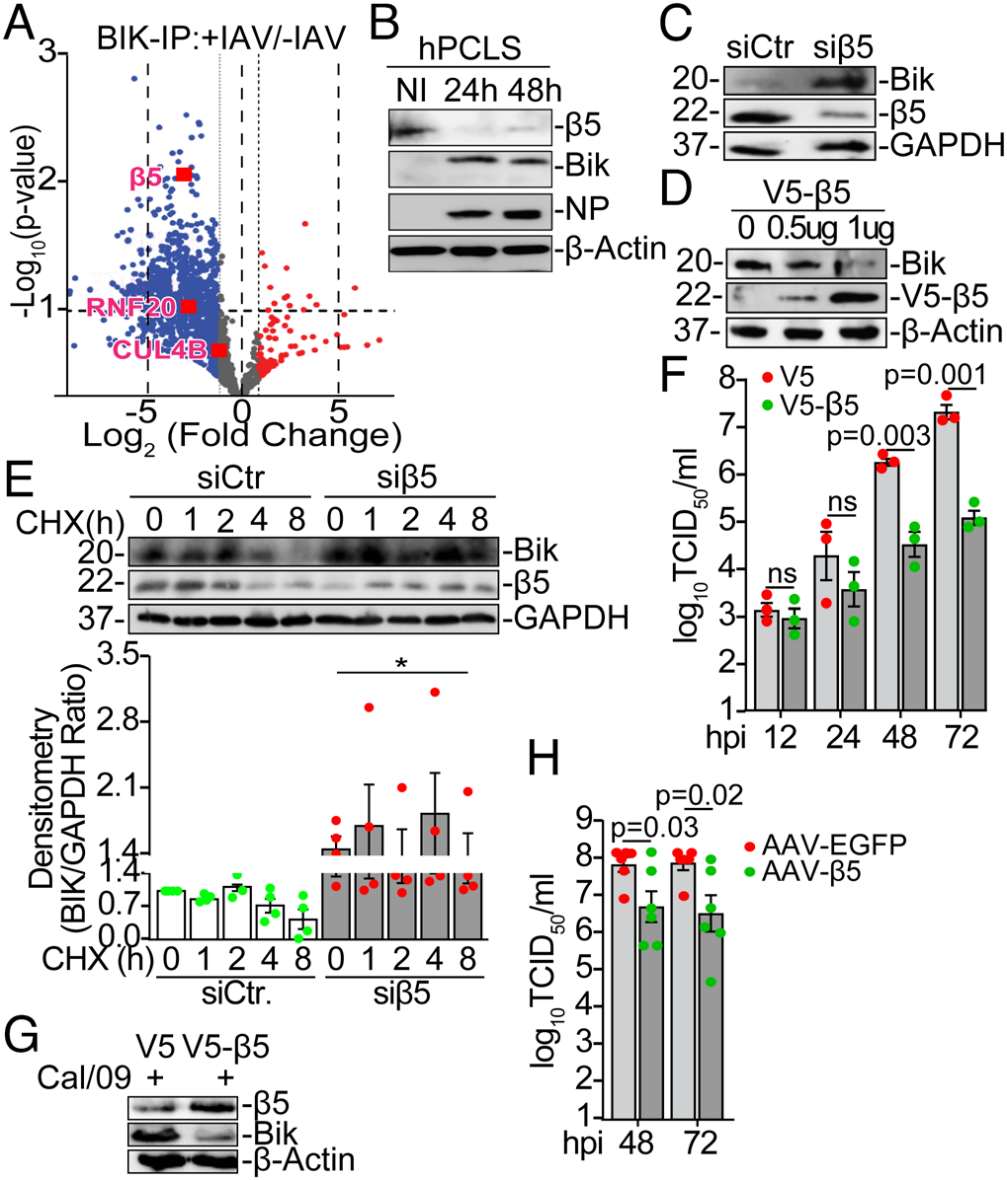
IAV-NP inhibits β5 to stabilize BIK and promote viral replication. (*A*) HEK293T cells were infected with vehicle or 1 MOI Cal/09. Protein lysates were immunoprecipitated with anti-BIK antibody and resolved using SDS-PAGE. An equal amount of BIK was pulled from vehicle- or Cal/09-infected cells and submitted for proteomics analysis. Protein bands were cut and subjected to Mass Spectrometry analysis. The Volcano plot was generated using GraphPad Prism software to identify the top BIK-interacting proteins increased or inhibited by IAV infection. n = 3/group. (*B*) Human precision-cut lung slices (hPCLS) were infected with mock (PBS, no infection (NI)) or Cal/09 for 24 and 48 h. Protein lysates were analyzed for the expression of β5, BIK, and NP. (*C*) NHBEs were transfected with siControl or siβ5. Forty-eight hours later protein lysates were analyzed for BIK and β5 levels. (*D*) NHBEs were transfected with 0 to 1 µg V5-β5. Forty-eight hours later protein lysates were analyzed for BIK and β5 levels. (*E*) A549 cells were transfected with siCtr or siβ5. Forty-eight hours later, cells were treated with 50 µg/mL cycloheximide (CHX) for the indicated time in hours (h). Protein lysates were analyzed for the expression levels of BIK and the half-life of BIK was compared. *P < 0.05. (*F* and *G*) A549 cells were transfected with V5 or V5-β5 followed by infection with 0.1 MOI Cal/09. (*F*) Viral titers in the apical washes were compared at the indicated time points using TCID_50_ assay. (*G*) Cell lysates were analyzed for BIK and β5 expression at 72 hpi. Two-tailed *t* test. (*H*) ALI-differentiated NHBE_A/A_ cultures were infected with 0.1 MOI Cal/09 and treated with 10^11^ genome copies/well of AAV-GFP or AAV-β5. Viral titers in the apical washes were compared at the indicated time points using TCID_50_ assay. n = 6/group. Two-tailed *t* test. Error bars: Mean ± SEM. Western blotting experiments were performed in triplicate, and representative images are presented.

BIK is a labile protein and can be stabilized by proteasome inhibitors. Proteasome inhibitors such as MG132 and bortezomib stabilize BIK in epithelial cells (30–33). Consistent with these reports, treatment of cells with MG132 (Fig. 5*D*) or bortezomib (*SI Appendix*, Fig. S6*C*) increased BIK protein levels. To determine whether IAV infection increases BIK levels by inhibiting BIK ubiquitination or by inhibiting degradation of ubiquitinated BIK, we immunoprecipitated BIK from IAV-infected and MG132-treated cells and analyzed for BIK ubiquitination. We found that, compared to vehicle-treated cells, both infection with IAV and treatment with MG132 inhibited the degradation of ubiquitin-conjugated BIK at 24 (Fig. 5*E*) and 48 hpi (*SI Appendix*, Fig. S6*D*). Interestingly, consistent with our finding that viral NP induced BIK protein level, expression of viral NP reduced the degradation of ubiquitin-conjugated BIK (Fig. 5*F*). Additionally, both IAV infection (Fig. 5*G* and *SI Appendix*, Fig. S6*E*) and NP expression (Fig. 5*H*) inhibited degradation of the K48-linkage polyubiquitin chain-conjugated BIK, suggesting involvement of the canonical proteasomal pathway. We hypothesized that IAV interferes with the proteasome to stabilize BIK. To test this, we infected HEK293T cells with mock (PBS) or Cal/09 (0.1 MOI). Cell lysates were immunoprecipitated with anti-BIK antibody and submitted for proteomic analyses (*SI Appendix*, Fig. S7*A*). Mass spectrometry analysis identified β5 among the top proteasomal proteins whose levels of association with BIK were significantly reduced in IAV-infected cells (Fig. 6*A* and *SI Appendix*, Fig. S7*B*). The 20S proteasome subunit β5 is encoded by the PSMB5 gene and contributes to the complete assembly of the 20S proteasome complex (39). β5, along with other β subunits, assemble into a proteolytic chamber for substrate degradation and contains “chymotrypsin-like” activity capable of cleaving peptides after large hydrophobic residues (39). To determine whether the expression of β5 was affected by IAV infection or viral NP expression we reprobed membranes and found that both infection with IAV (Fig. 5*A*) and overexpression of viral NP (Fig. 5*C*) inhibited β5 expression, suggesting that IAV-NP inhibits degradation of ubiquitin-conjugated BIK by the catalytic subunit of the 20S proteasome. In human precision-cut lung slices (hPCLS), Cal/09 infection suppressed β5 and increased BIK protein levels at 24 and 48 hpi (Fig. 6*B*). Immunostaining confirmed reduced β5 expression in IAV-infected hPCLS (*SI Appendix*, Fig. S7*C*). Depletion of β5 in NHBEs increased BIK protein level (Fig. 6*C*), whereas its overexpression reduced BIK levels (Fig. 6*D*). Additionally, siRNA-mediated β5 silencing stabilized BIK in cycloheximide-treated cells (Fig. 6*E*).

### Treatment with β5 Inhibits IAV Replication in the AECs and Protects Mice from IAV-Induced Morbidity and Mortality.

**Fig. 7.**
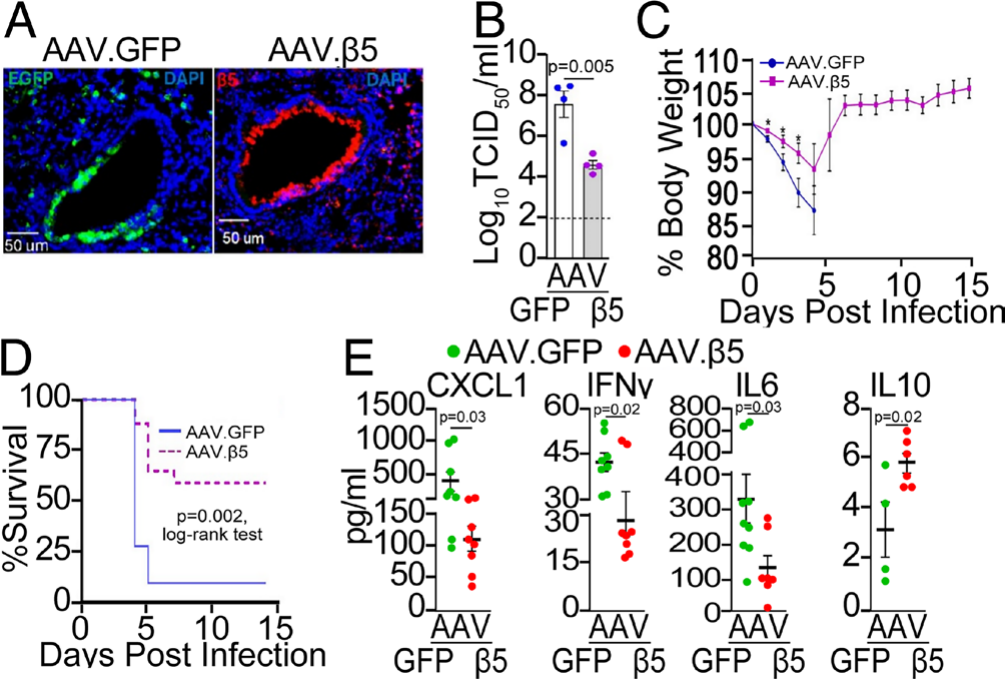
β5 protects mice from IAV-induced morbidity and mortality. WT C57BL/6 mice were instilled intranasally with 10^11^ GC of AAV6.2-EGFP or AAV6.2-β5. Mice were infected with 250 pfu PR/8 on day 5 post-AAV treatment. On day 5 post PR/8 infection, (*A*) paraffin-embedded lung tissues were immunostained for EGFP or β5 and analyzed using fluorescent microscopy and (*B*) lung viral load was measured using TCID_50_ assay. (*C* and *D*) Mice were weighed daily and monitored for survival over a period of 14 dpi. n = 10 to 13/group, *P < 0.05. (*E*) Inflammatory cytokine and chemokine levels in the lung homogenates analyzed at 5 dpi using Luminex multiplex assay. *t* test; n = 4-10/group. Error bars: Mean ± SEM.

Consistent with the above findings, overexpression of V5-β5 in vitro resulted in a significant reduction in viral titers 48 and 72 hpi (Fig. 6*F* and *SI Appendix*, Fig. S7*D*) and suppressed BIK protein levels (Fig. 6*G*). Similarly, treatment of well-differentiated NHBE_A/A_ with β5-expressing adeno-associated viral vector (AAV), AAV6.2-β5, diminished viral load significantly (Fig. 6*H*) and suppressed BIK expression (*SI Appendix*, Fig. S7*E*). These data suggest that IAV-NP hijacks β5 to block degradation of ubiquitin-conjugated BIK by the proteasome machinery, thereby stabilizing BIK for efficient viral replication. In vivo, administration of AAV6.2-β5 (Fig. 7*A*) reduced NP expression in the airways (*SI Appendix*, Fig. S8*A*), decreased lung viral load (Fig. 7*B*), and protected mice from IAV-induced weight loss (Fig. 7*C*) and mortality (Fig. 7*D*). Furthermore, AAV6.2-β5-treated mice exhibited significantly lower levels of CXCL1, IL-6, IFN-γ, and higher levels of the anti-inflammatory cytokine IL-10 in lung homogenates compared to AAV6.2-EGFP-treated controls (Fig. 7*E*). Sex-stratified analysis confirmed that AAV6.2-β5 treatment conferred protection against weight loss (*SI Appendix*, Fig. S8*B*) and mortality (*SI Appendix*, Fig. S8*C*) in both male and female mice, demonstrating that treatments with β5 conferred protection regardless of sex. Collectively, these data support the therapeutic potential of β5 for inhibiting IAV replication in AECs and mitigating virus-induced morbidity and mortality.

### BIK Interacts with Viral NP to Disrupt Bcl-2/NP Interaction and Promote Viral Protein Accumulation.

**Fig. 8.**
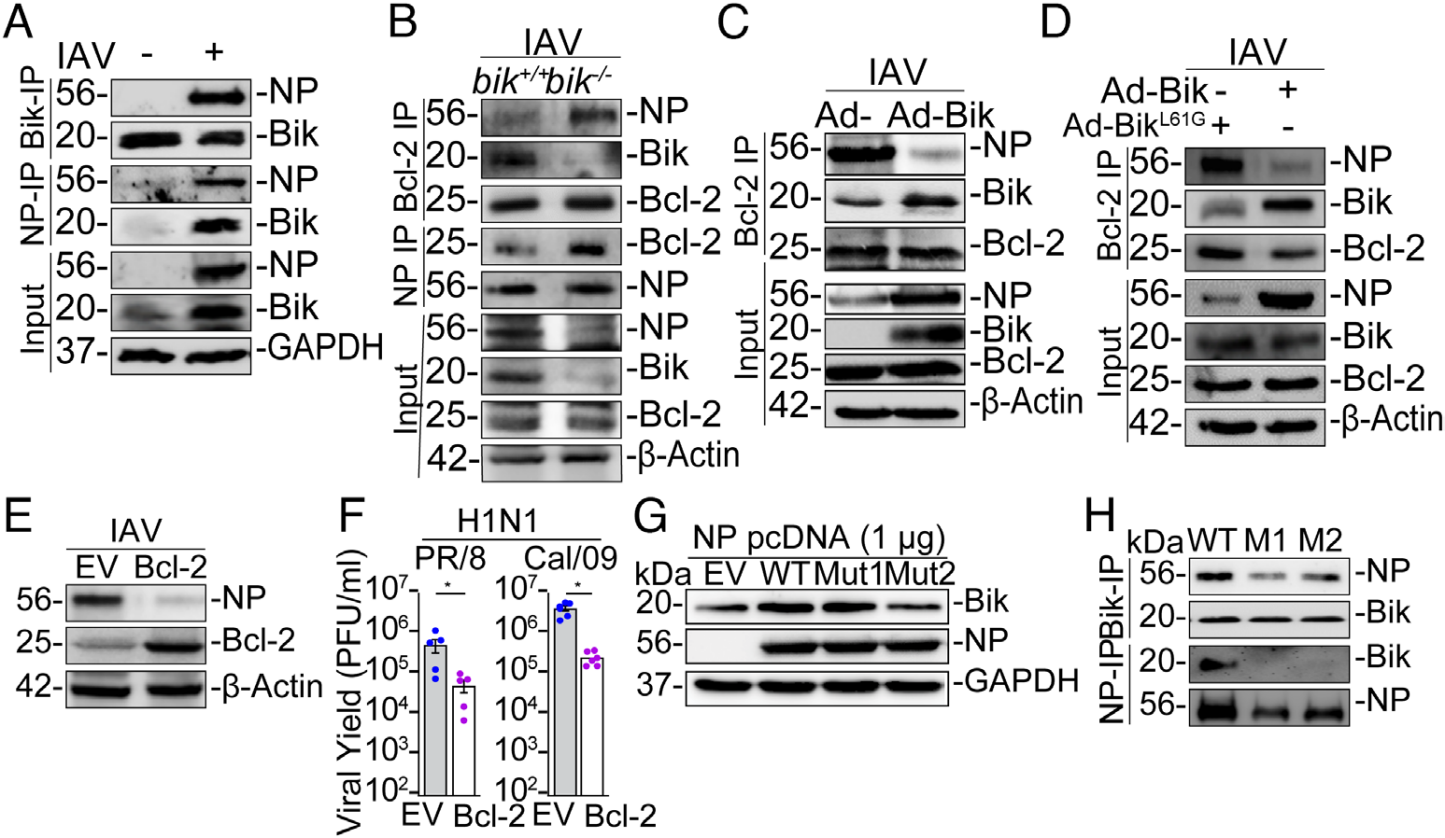
*BIK/NP* interaction stabilizes vRNP. (*A*) HAECs were infected with the vehicle or 0.1 MOI PR/8 and protein lysates were treated with RNase A. The NP and BIK immunoprecipitates (IPs) were analyzed for BIK and NP levels. (*B*) *bik*^+/+^ and *bik^−/−^* MAECs were infected with 1 MOI PR/8. Protein lysates were treated with RNase A. NP and Bcl-2 IPs were analyzed. (*C*) HAECs were infected with 0.1 MOI PR/8 and 24 h later treated with empty adenoviral vector (Ad-) or 50 MOI Ad-BIK. Protein lysates were treated with RNase A. Bcl-2 IP was analyzed for NP and BIK protein levels. (*D*) HAECs were infected with 0.1 MOI PR/8 and 24 h later with 50 MOI Ad-BIK^L61G^ (with impaired interaction with Bcl-2) or Ad-BIK^WT^. Protein lysates were treated with RNase A. Bcl-2 IPs were analyzed for NP and BIK protein levels. (*E*) HEK293T cells were transfected with EV or Bcl-2 expression vector and infected with 0.1 MOI PR/8 24 h later. Protein lysates were analyzed for the level of NP and Bcl-2 24 hpi. (*F*) HEK293T cells transfected with EV or Bcl-2 expression vector and infected with 0.1 MOI of the indicated IAV strains. Virus titers were compared in the apical washes using plaque assay at 72 hpi. *P < 0.05. (*G* and *H*) HEK293T cells were transfected with 1 µg EV, WT-, Mut1 (E46A, K48A)-, or Mut2 (D101A)-NP expressing constructs. (*G*) Cell lysates were analyzed for BIK and NP levels. (*H*) Protein lysates were immunoprecipitated with anti-NP or anti-BIK antibodies and analyzed for BIK/NP interaction. Western blotting experiments were performed in triplicate, and representative images are presented. Error bars: Mean ± SEM.

Earlier studies showed that increased levels of Bcl-2 impairs IAV replication (17, 18, 40). BIK is a Bcl-2-interacting protein (41) and promotes viral replication (Fig. 1 *B*, *C*, *H*, and *J*) (19). NP is highly conserved viral protein and interacts with components of the vRNP such as PB1 and PB2 (42), and NS1 (43). Therefore, to investigate whether BIK interacts with components of vRNP, we coexpressed Flag-NP, -PA, -PB1, -PB2, or -NS1 plasmids with Ad-BIK in HEK293T cells. Cells were detergent lysed, immunoprecipitated with anti-Flag antibody, and analyzed for BIK protein by Western blot. BIK copurified with NP but no other components of vRNP or NS1 (*SI Appendix*, Fig. S9*A*). Similarly, BIK interacted with viral NP in both PR/8-infected (Fig. 8*A*) and Cal/09-infected (*SI Appendix*, Fig. S9*B*) HAECs. Immunostaining analysis of HAECs infected with PR/8, HKx31, or Cal/09 strains (*SI Appendix*, Fig. S9*C*) or transfected with NP expressing plasmid (*SI Appendix*, Fig. S9*D*) showed BIK-NP colocalization. We also observed strong Bcl-2/NP interaction in *bik^−/−^* MAECs compared to weak Bcl-2/NP interaction in *bik^+/+^* MAECs (Fig. 8*B*). Mechanistically, increased BIK expression via Ad-BIK, compared to the EV control (Ad-), reduced the Bcl-2/NP interaction (Fig. 8*C*), suggesting that BIK dissociates NP from Bcl-2. Our previous data showed that while the WT BIK interacts strongly with Bcl-2, a Leu61/Gly mutation in the BH3 domain of BIK impairs its interaction with Bcl-2 (41). Consistent with these findings, reconstituting BIK in *bik^−/−^* MAECs using Ad-BIK disrupted the Bcl-2/NP interaction, whereas restoring the Leu61/Gly mutant BIK (Ad-BIK^L61G^) did not affect the Bcl-2/NP interaction (Fig. 8*D*). These interactions occur in lysates treated with RNase A, suggesting that the BIK/NP and Bcl-2/NP interactions are protein–protein interactions not mediated by viral RNA. Next, to investigate whether an increased level of Bcl-2 affects viral replication, we transfected HEK293T cells with EV or Bcl-2 expression plasmid and analyzed the viral NP level and titer at 72 hpi. The viral NP levels (Fig. 8*E*) and viral loads (Fig. 8*F* and *SI Appendix*, Fig. S9*E*) were diminished in Bcl-2-overexpressing cells. Together, these data suggest that the NP/Bcl-2 interaction leads to degradation of NP to inhibit viral replication, whereas BIK disrupts the Bcl-2/NP interaction, thereby increasing NP levels for efficient viral replication.

IAV-NP consists of head and body domains, with a RNA-binding groove between them (44). The body domain interacts with other vRNP components (42, 44–46). To determine whether the NP body domain is essential for BIK/NP interaction and BIK stabilization, we created NP mutants with altered amino acid regions within the conserved body domain using nested PCR (*SI Appendix*, Fig. S9*F*). While the WT-NP and mutant E46A/K48A-NP (Mut1) induced BIK, the D101A-NP (Mut2) did not affect the level of BIK protein (Fig. 8*G*). Immunoprecipitation assays (Fig. 8*H* and *SI Appendix*, Fig. S9*G*) showed that WT-NP and BIK interacted strongly, whereas mutants E46A/K48A (Mut1) and D101A (Mut2) resulted in weaker interaction with BIK, suggesting that D101 in the body domain of NP is crucial for the BIK/NP interaction.

## Discussion

We demonstrate that reduced BIK levels in primary AECs decrease viral replication, while targeted BIK expression in mouse airways increases viral load, inflammation, and mortality. In humans, the rs738276 *BIK* promoter SNP modulates BIK expression, influencing viral replication and inflammation in primary AECs. Critically, the AA variant, associated with elevated BIK, correlates with severe influenza. Mechanistically, IAV-NP suppresses proteasomal β5, stabilizing BIK. BIK interacts with NP, and β5 treatment reduces BIK and viral replication. These findings identify BIK as a key host factor impacting influenza severity and suggest rs738276 as a potential marker for personalized prevention and treatment.

While our study focused on limited IAVs, our findings suggest a broader role for BIK in viral replication, potentially through a conserved mechanism. In mice, airway-specific BIK overexpression (using the CCSP promoter in a *bik^−/−^* background) increased lung viral load, inflammation, and susceptibility to lethal IAV dose, while shBIK-mediated BIK reduction decreased viral load and protected mice against severe outcomes. Given the airway epithelium’s role in IAV replication (47, 48), reducing virus burden there may enhance host immune clearance. The high viral load and associated inflammation observed in some of the mouse studies are consistent with previously published findings using similar models (29, 49), where rapid disease progression and mortality within 5 dpi are commonly observed. We acknowledge the inherent biological variability in the in vivo experiments. Even with highly controlled conditions, individual animals can respond differently to infection, leading to variations in viral load, as observed in Fig. 2*E* and *SI Appendix*, Fig. S3*F*. These variations can arise from subtle differences in factors such as age, sex, weight, immune status, or even gut microbiome composition. Furthermore, minor experimental variations, including the precise handling of the virus stock, or timing of infections can also contribute to variability in the observed viral titers. Sex did not significantly affect IAV infection outcomes in this study.

NHBE_AA_ exhibit higher *BIK* mRNA and protein levels compared to NHBE_GG_, with NHBE_AG_ showing intermediate levels, consistent with our prior findings (34). Reduced viral load in NHBE_G/G_, and *bik*-deficient cells, suggest that elevated BIK promotes replication. Given the rs738276 region’s homology to IRF-1 binding site, and IRF-1’s preferential binding to G allele to repress BIK (34), the AA variant likely contributes to elevated BIK, thereby enhancing influenza disease severity (*SI Appendix*, Fig. S10*B*). SNPs like rs738276 can impact gene expression through diverse mechanisms (50–54), affecting protein levels and disease susceptibility (55–58), suggesting that altered splicing may also contribute BIK regulation.

BIK, a founding member of BH3-only proteins (59), is a proteasome target (30–33, 41). While BIK ubiquitination and degradation are stress-dependent (31), IAV infection inhibits BIK’s ubiquitin-dependent proteasomal degradation, as does viral NP, suggesting a specific role of NP in BIK stabilization. Our data suggest that the BIK/NP interaction may alter BIK ubiquitination, or NP directly blocks proteasomal activity. Indeed, our proteomic and in vitro analyses identified β5, a critical BIK-interacting 20S proteasomal subunit (39, 60), suppressed by IAV. Since β5 possesses chymotrypsin-like activity crucial for substrate degradation (39), we propose IAV-NP inhibits β5, blocking ubiquitin-conjugated BIK degradation and stabilizing BIK for viral replication.

Our data demonstrates that IAV actively induces BIK for productive infection. The reciprocal Bcl-2/NP and Bcl-2/BIK interactions suggest competition for the same Bcl-2 binding site. Consistent with prior work (17, 18), Bcl-2 inhibits IAV replication, while here we found that IAV-induced BIK disrupts the Bcl-2/NP interaction, potentially promoting vRNP transcription/replication by freeing NP from Bcl-2. NP, a crucial IAV structural protein (61), interacts with BIK *via* its body domain, a region critical for vRNP function (42, 44, 45). Our previous data showed that while the WT BIK interacts strongly with Bcl-2, a L61G mutation in the BH3 domain impairs its interaction with Bcl-2 (41). Unlike the WT BIK, however, the L61G mutant fails to efficiently dissociate NP from Bcl-2. While the WT NP interacted with and induced BIK, the body domain-mutated NP failed to interact with or induce BIK, indicating the body domain’s significance for BIK/NP interactions. We propose that IAV-induced BIK interacts with the NP body domain to promote vRNP transcription/replication (*SI Appendix*, Fig. S10*A*). These BIK/NP interaction sites offer potential therapeutic targets.

This study provides an in-depth insight into how host BIK impacts viral protein stability and replication. Disrupting BIK/NP interactions or enhancing β5 activity to promote BIK degradation could offer novel antiviral strategies. *BIK* SNP’s association with influenza severity reveals host genetic risk factors, suggesting its use as a screening marker for personalized treatment. These findings offer promising avenues for improved influenza prevention and treatment.

This study utilized the HKx31 strain, a reassorted virus with HA and NA derived from HK/68 (H3N2) and six gene segments from PR/8, as well as the WT HK/68 in specific experiments. This inclusion allows us to demonstrate that BIK’s role in viral replication is not limited to the reassorted HKx31 strain and extends to the parental HK/68 virus. By using both strains, we strengthen the conclusion that BIK is important for replication of different viruses, including those derived from the HK/68 H3N2 lineage, and suggests a broader relevance of BIK in H3N2 virus replication beyond the specific genetic background of HKx31. However, we acknowledge that further studies using a wider range of currently circulating H3N2 strains are necessary to definitively establish the full scope of BIK’s influence across diverse viral backgrounds. Furthermore, the viruses used in this study, including PR/8, Cal/09, and those with the HK/68 HA/NA, share the NP derived from the 1918 pandemic strain. While our data strongly suggest a link between BIK and IAV replication independent of HA/NA, the use of viruses with a non-1918 NP would definitively confirm this observation and rule out any potential influence of the 1918 NP on BIK-mediated effects. Future studies should therefore investigate BIK’s role in the replication of a broader panel of currently circulating IAV strains, including more recent H3N2 isolates and other subtypes. Critically, these studies should include viruses with diverse NP backbones to definitively establish the generality of the IAV–BIK–β5 axis and its independence from the 1918 NP. Such experiments would solidify the clinical relevance of targeting BIK or β5 for therapeutic intervention against a wider range of influenza viruses. The studies using the human cohort also have key limitations, including the small size and demographic limitations, which warrant larger studies. Furthermore, we did not explore all BIK regulatory pathways, and preclinical studies are needed to evaluate the safety and efficacy of targeting β5 or BIK.

In conclusion, this study reveals a critical IAV–BIK–β5 axis where IAV-stabilized BIK, through β5 suppression, enhances viral replication and severity. The genetic link between BIK expression and human influenza outcomes, coupled with the BIK/NP interaction, highlights BIK and β5 as promising targets for novel antiviral therapies.

## Materials and Methods

Key reagents or resources and detailed methodologies are provided in the supplementary materials. This study utilized the FLU09 cohort, previously described for participant recruitment and data collection, and involved WT C57BL/6, *bik^−/−^*, *noxa^−/−^*, and airway-specific BIK-transgenic mice. Human BIK knockout cell lines were generated using LentiCRISPRv2, while human precision-cut lung slices were prepared from healthy donor lung tissue following established protocols. Cytokine analysis was performed on lung homogenates and cellular supernatants using Luminex-based multiplex assays. NP mutant plasmids were created by nested-PCR. SNP genotyping for rs738276 was conducted on DNA from NHBEs and PBMCs using TaqMan genotyping. Proteomic analysis of BIK immunoprecipitates was performed after SDS-PAGE separation to identify interacting proteins. All sample manipulations and mice experiments were approved by the Institutional Review Board and IACUC and performed in strict accordance with the institutional guidelines. For further methodological details, please see *SI Appendix*.

## Supplementary Material

Appendix 01 (PDF)

## Data Availability

All study data are included in the article and/or *SI Appendix*.
