# Supplementary material for: BIK polymorphism and proteasome regulation unveil host risk factor for severe influenza
Source: Proc Natl Acad Sci U S A. 2025 Jul 8;122(28):e2424367122. doi: 10.1073/pnas.2424367122 (PMC12280918; doi:10.1073/pnas.2424367122)
Supplement: Supplementary file 1 — Appendix 01 (PDF) [file pnas.2424367122.sapp.pdf]

Supplemental Information

BIK Polymorphism and Proteasome Regulation Unveil Host Risk Factor for Severe Influenza

Sourabh Soni[1], Soner Yildiz[2,3], Emma Kaitlynn Allen[4], Hans Petersen[5], Mark Peeples[6], Sara El Zahed[2,3], Lorena Rosas[1], Vandana Anang[1], Laura Antonescu[1], Richard Seonghun Nho[1], Ana Lucia Mora[1], Jeffrey Craig Horowitz[1], Mauricio Rojas[1], Rafael Andrés Medina[12], Paul Glyndwr Thomas[3], Adolfo García-Sastre[2,3,7,8,9,10], Yohannes Tesfaigzi[11], Yohannes Afework Mebratu[1]

Materials and Methods

Study cohort

The protocol for the recruitment of participants and collection of data in the FLU09 cohort has been previously described (1). Briefly, participants were included if they met the clinical criteria for influenza virus infection at enrollment or were asymptomatic household contacts of a confirmed influenza case. Exclusion criteria included refusal of nasal lavage, a history of receiving immunoglobulin or blood products within 3 months of enrollment, and any condition that could cause undue harm or prevent the participant from following the study protocol. Inclusion and exclusion criteria were established before enrollment of the participants. This study was conducted in accordance with 45 CFR 46 and the Declaration of Helsinki. The study was approved by the Institutional Review Boards of St. Jude Children's Research Hospital and the University of Tennessee Health Science Center/Le Bonheur Children's Hospital. The study obtained written, informed consent from participants' parents/guardians, along with written assent from age-appropriate subjects at enrollment. Index cases provided nasal swabs, nasal lavages, and blood on enrollment (Day 0) and Days 3, 7, 10, and 28. Household contacts provided nasal swabs on Days 0, 3, 7, and 14, and blood and nasal lavages on Days 0 and 28. The study population comprised predominantly African American individuals (80%), with 20% Caucasian participants. Collected metadata included daily self-reported symptoms ranked on a visual analog scale, categorized into systemic (fever, fatigue, headache, body ache, chills, lethargy, and irritability), upper respiratory (sore throat, stuffy nose, earache, and sinus pressure), lower respiratory (cough, shortness of breath, and wheezing), and gastrointestinal symptoms (nausea, vomiting, and diarrhea). FLU09 participants were classified into mild and severe patient groups based on their total peak symptom score during infection, with severe patients having a total peak score greater than 600. Severe lower respiratory tract symptom status was defined by the presence of shortness of breath and wheezing.

Mice

Wild-type C57BL/6 mice were procured from The Jackson Laboratory (Bar Harbor, ME, USA). The *bik*$^{+/-}$ and *noxa*$^{+/-}$ mice on a C57BL/6 background were generously provided by Andreas Strasser (Walter and Eliza Hall Institute, Melbourne, Australia). *bik*$^{+/+}$, *bik*$^{-/-}$, and *noxa*$^{-/-}$ littermates were bred from the

respective heterozygote mice under specific pathogen-free conditions and genotyped accordingly. Conditional and airway-specific overexpression of BIK was achieved through a crossbreeding strategy involving two lines of transgenic mice: CCSP-reverse tetracycline-responsive transactivator (CCSP-rtTA) mice, harboring the rtTA gene under the control of the CCSP gene promoter, and $TetO_7$-BIK mice, containing the TetO and minimal cytomegalovirus (CMV) promoter along with the BIK transgene. $TetO_7$-BIK mice were generated at the MD Anderson Genetically Engineered Mouse Facility (Houston, TX, USA) following standard protocols approved by the Institutional Animal Care and Use Committee (IACUC). These mice were then bred with CCSP-rtTA mice to obtain the desired genotype. To generate airway-specific BIK transgenic mice on a $bik^{-/-}$ background, the BIK transgene mice were crossed with $bik^{-/-}$ mice. Conditional expression of BIK in the lung was induced by administering doxycycline-containing water (400 mg/l) to the transgenic mice. Both male and female animals were used in the study. All mice were housed individually in isolated cages under specific pathogen-free conditions. Following a 14-day quarantine period, mice were acclimatized for an additional 8 days before being included in the experimental protocol at 6-8 weeks of age. Ethical approval for all experiments was obtained from the IACUC of Brigham and Women's Hospital and The Ohio State University, facilities accredited by the Association for Assessment and Accreditation of Laboratory Animal Care International.

Cells

Mouse airway epithelial cells (MAECs) were isolated from the trachea of 6 to 8-week-old WT C57BL/6 mice. The cells were cultured either on plastic plates or Transwell membranes (Corning, New York, NY, USA) after seeding with a density ranging from 4×10^4 to 9×10^4 cells per plate. The primary normal human bronchial epithelial cells (NHBEs) were obtained from de-identified donors (n=50) through a collaboration with Dr. Mark Peeples at Nationwide Children's Hospital and Dr. Yohannes Tesfaigzi at Harvard Medical School. These cells were then genotyped to determine the *BIK* rs738276 SNP genotype. NHBEs were selected for subsequent experimental studies based on the following pre-defined criteria: 1) Passage Number: Cells were used at passage 2 or 3 to minimize potential phenotypic drift. 2) Differentiation Capacity: Only NHBEs demonstrating the ability to differentiate in air-liquid interface (ALI) culture were included. To ensure balanced representation across genotypes and minimize potential

confounding factors, we aimed to select 10 NHBE lines for each *BIK* genotype for use in the study. These lines were selected to be age-matched and balanced for sex whenever possible within the available pool of genotyped donors. These cells were allowed to differentiate as previously described (2) (3). Briefly, NHBEs were seeded onto collagen-coated transwell inserts with 0.4 um pores and allowed to grow to confluency while being fed on both apical and basolateral sides with ALI culture medium (complete SAGM media) (Lonza, Walkersville, MD, USA) containing TGFβ antagonist, BMP4 antagonist, WNT agonist, and ROCK inhibitor (StemCell Technologies Canada Inc., Vancouver, British Columbia, Canada) in a 37°C incubator supplied with 5% CO_2. When the cultures reached confluency, the proliferation medium with inhibitors was removed from the cultures and fed with differentiation medium, PneumaCult-ALI, below the filter and exposing the cells to the air. The medium was changed every second day until the cells differentiated into airway epithelium with ciliated cells and mucus production. NHBEs that did not differentiate in ALI were not included in the study. Additionally, immortalized human airway epithelial cells (HAECs), AALEB cells, were obtained from Dr. Scott Randell (University of North Carolina, Chapel Hill, NC, USA). HAECs were maintained in complete BEGM media (Lonza) in a 37°C incubator. Other sets of HAECs were ALI-differentiated on Transwell membranes (Corning) as previously described (4). Human lung epithelial cells (A549), human embryonic kidney cells (HEK293T), and Madin Darby canine kidney cells (MDCK) were procured from American Type Culture Collection (ATCC, Manassas, VA, USA) were cultured in Dulbecco's Modified Eagle Medium (DMEM) supplemented with 10% fetal bovine serum (FBS) and 1% penicillin/streptomycin (Gibco, Thermo Fisher Scientific, Waltham, MA, USA). Transfection of cells with plasmid DNA was performed using TransIT-2020 transfection reagent (Mirus, Madison, WI, USA). Prior to experimental use, cells were routinely screened for mycoplasma contamination to ensure data integrity.

Generation of *BIK* knockout cells using CRISPR-Cas9 genome editing

Human *BIK* knockout cell lines were generated utilizing LentiCRISPRv2 obtained from GenScript USA Inc. (Piscataway, NJ, USA). The plasmids were transduced into HEK293T cells using TransIT-2020 transfection reagent (Mirus) to produce lentivirus. After 48 hours post-transduction, the viral supernatant was collected and filtered through 0.45 μm filters (Millipore, Burlington, MA, USA). HEK293T cells were

infected with the lentivirus, and subsequent antibiotic selections were performed to enrich cells with successful integration of the CRISPR/Cas9 system targeting the *BIK* gene. Following antibiotic selection, individual cells were seeded onto 96-well plates to facilitate clonal selection. Clones that emerged were then subjected to screening for *BIK* gene knockout using Western blot analysis.

Air-liquid interface (ALI) culture

NHBE cells were seeded onto 12-well Transwells (Corning) coated with 0.3 mg/ml human collagen type IV (Sigma-Aldrich, St. Louis, MO, USA) in expansion media (PneumaCult-Ex Plus, STEMCELL Technologies Inc.). The cells were cultured in this medium for one week until they formed a confluent monolayer. Subsequently, the cells were transitioned to an ALI culture system using a differentiation media (PneumaCult ALI, STEMCELL Technologies Inc.) for a duration of three weeks. During the ALI culture period, the medium was refreshed three times a week to maintain cell viability and integrity. The cells were maintained at a temperature of 37°C with a 5% CO_2 atmosphere to mimic physiological conditions conducive to airway epithelial cell growth and differentiation.

Human precision-cut lung slices (hPCLS) preparation

Human lung tissue was obtained from healthy donors by the Comprehensive Transplant Center (CTC) Human Tissue Biorepository at The Ohio State University with Total Transplant Care Protocol informed consent and research authorization form. CTC operates in accordance with NCI and ISBER Best Practices for Repositories. hPCLS were prepared according to previously established protocols (5) (6). Briefly, transplant-rejected lungs were infused with warm (37°C) 3% of UltraPure™ Low Melting Point Agarose (Thermo Fisher Scientific) in sterile medium (DMEM, Gibco) *via* a visible bronchus. Lung segments were transferred into a tube with phosphate-buffered saline (PBS) and cooled on ice for 30 min to allow gelling of the agarose. Approximately 1cm^3 of human lung tissue was embedded and sliced at 400µm of thickness using a vibrotome (0.10-0.30 mm/s; Leica VT 1200, Nussloch, Germany). The slices were incubated at 37°C in a tissue incubator with 5% CO_2 and then washed in sterile medium (DMEM/F-12, Gibco) three times to remove agarose. This was followed by overnight incubation in DMEM/F-12 medium (Gibco) with 10% fetal bovine serum (FBS, Gibco) and 1% penicillin/streptomycin (Gibco) prior to studies being performed. hPCLS were infected with IAV Cal/09 (1*10^6 pfu) in serum-

free infection media containing 3ug/ml TPCK-Trypsin for approximately 1.5 hours (intermittent shaking). After removing the infection media, hPCLS were washed with PBS and fresh complete media was added followed by 48/72hrs incubation in 5% CO_2 before processing for downstream experiments.

Viruses, viral growth, and titer determination

Various influenza virus strains, including A/California/04/2009 (Cal/09, H1N1), A/Puerto Rico/8/1934 (PR/8, H1N1), A/Hong Kong/1/1968 (HK/68, H3N2), and A/HKx31 (HKx31, H3N2) were kindly provided by Dr. Adolfo García-Sastre at the Icahn School of Medicine at Mount Sinai, NY, USA. IAV (H1N1) PR/8/34 strain was propagated in embryonated chicken eggs and titrated on MDCK cells as described (7). Infectious virus yields were analyzed from the apical washes and cellular supernatants collected 48- or 72-hours post-infection (hpi) *via* plaque assay as described (8). Primary MAECs were allowed to differentiate in ALI and infected from apical side with mock or 0.1 MOI of the virus for 1 hour followed by washing with PBS. Further, fresh medium was added, and cells were incubated at 37°C. Infectious virus yields were analyzed from cell supernatants using plaque assay as described (8). Briefly, monolayers of MDCK cells were cultured overnight in 96-well culture plates in DMEM containing 10% FBS and 1% penicillin-streptomycin and infected with 10-fold serial dilutions of virus suspension made in media containing TPCK-trypsin (3µg/ml) for 1 hour at room temperature (RT). Cells were then covered with warmed 2x DMEM and 2% purified agar. The agar medium was allowed to solidify at RT and incubated for 3 days at 37°C to promote plaque development. Immediately, prior to plaque analysis, the solidified agar was removed, and cells were fixed with 10% formalin and stained with a 1% crystal violet solution made in 20% ethanol. Plaques were counted, and the virus titer was expressed as plaque-forming unit (pfu)/ml.

Viral titers in lung tissues and median tissue culture infectious dose ($TCID_{50}$) assay

Mouse lungs were harvested at 5 dpi and homogenized using Precellys lysing kit and tissue homogenizer (Bertin Technologies, Montigny-le-Bretonneux, France) following previously established protocols (9). Subsequently, the supernatants were collected and divided into aliquots for further analysis. Virus titration was performed immediately after sample preparation. $TCID_{50}$ assay was employed for viral quantification. Confluent monolayers of MDCK cells in 96-well plates were inoculated with 10-fold dilutions of the

samples in FBS free media containing 2μg/ml TPCK-trypsin, with 9 wells per dilution, and incubated for 3 days. Cells were fixed with 4% paraformaldehyde for 15 min and stained with a 0.25% crystal violet solution made in 20% methanol. Wells displaying positive cytopathic effects were counted, and the $TCID_{50}$ titer was determined by interpolating the results using the Reed-Muench method (10), as previously described (11).

Processing of murine lung tissues

Following euthanasia, murine lungs were perfused with PBS through the right ventricle to rinse away remaining blood. Both lungs were removed, snap frozen in liquid nitrogen, and stored in -80°C. In other cohorts of mice, lungs were inflated with 10% neutral buffered formalin, embedded in paraffin, and sectioned. 5 μm thick formalin fixed paraffin-embedded lung sections were stained with hematoxylin and eosin (H&E) to assess inflammation-associated lung damage. Histopathological evaluation of lung tissues was conducted by a certified pathologist in a blind manner. Images of lung sections were captured at 20x magnification and multiple random fields were analyzed per animal. Image acquisition and analyses were performed by observers blinded to the groups using VS200 Slide Scanner microscope and OlyVIA V3.4.1 software (Olympus Corporation, Tokyo, Japan) and ImageJ v1.53t software (NIH, MD, USA). Inflammatory changes in the lung were assessed using a semi-quantitative scoring system based on the relative degree of inflammation and tissue damage, as previously described (9) (12). The scoring system included parameters such as peribronchiolar and bronchial infiltrates, bronchiolar and bronchial luminal exudates, perivascular infiltrates, parenchymal pneumonia, and edema. Each parameter was graded on a scale of 0-4, with 0 indicating absence of inflammation and 4 indicating severe inflammation. Specifically, scores were assigned as follows: 0 for absent, 1 for slight, 2 for mild, 3 for moderate, and 4 for severe. The cumulative scores of inflammatory infiltration, degeneration, and necrosis provided the total score per animal. The average score of mice in each experimental group was calculated to determine the overall inflammation score for that group.

Luminex multiplex assay

Lung homogenates were prepared from virus treated mice 5 dpi and homogenized with the help of Precellys lysing kit and tissue homogenizer (Bertin Technologies). Also, cellular supernatants were

collected from NHBEs infected with viruses at 48 and 72 hpi. The lung homogenates and cellular supernatants were sent to Clinical Translational Science Shared Resource (CTSSR), The Ohio State University to analyze the changes of cytokines using a Luminex-based multiplexing assay employed using Luminex 200 (Luminex Corporation, Austin, TX, USA). The customized 10 cytokines mouse and 12 cytokines human assay kits were purchased from BioTechne R&D Systems (Minneapolis, MN, USA). All reagents were prepared following the manufacturer's instruction. The assays were performed in 96-well plates provided with the kits. The standards were reconstituted in 1:3 serial dilutions undertaken to generate a 7-standard concentration set and a blank using the diluent solution. 50μl of standards, diluent solution (blank) or 50μl of lung homogenates and cellular supernatants was added to each well with duplication, followed by the addition of the 50μl of diluted Microparticle Cocktail containing magnetic beads coated with the 29 capture antibodies, respectively. The plates were covered with foil and incubated for 2 h at room temperature (RT) on an orbital plate shaker at 800 rpm. The beads were protected from light throughout the course of the assay. At the end of this incubation, the plates were placed on a magnetic plate and sat for 2 minutes. The plates were washed three times by adding 100 μl of washing buffer, followed by adding 50μl of diluted Biotin-Antibody Cocktail to each well. The plates were covered and incubated for 1 hour at RT on the shaker at 800 rpm. After three washings, 50μl of diluted Streptavidin-PE was added to each well and incubated for 30 minutes at RT on the shaker at 800 rpm. After three washings, 100μl of washing buffer to each well and incubate for 2 minutes at RT on the shaker at 800 rpm. The plates were read using the Luminex 200 under the XPONENT software. The raw data was measured as mean fluorescence intensity (MFI) and the concentration (pg/ml) of each analyte for each sample was calculated using the standard curve generated for each analyte from the 7 standards and a blank, and the results were exported as a CSV/Excel file for statistical analysis.

Plasmids, vectors, adenoviral constructs, and reagents

Adenoviral expression vectors encoding BIK and BIKL61G were generously provided by Gordon Shore (McGill University, Montreal, Quebec, Canada), as described previously (13). We procured adeno-associated viral vector (AAV) 6.2LP-β5 and AAV6.2CP-GFP with serotype 6.2 capsid from VectorBuilder, Inc. (Chicago, IL, USA), and validated its transduction efficiency in airway epithelial cells (AECs) and in

the lungs of mice. The plasmids used in this study included Empty Vector and WT Bcl-2, which were kindly provided by Dr. Yohannes Tesfaigzi (Brigham and Women's Hospital, Boston, MA, USA). Transfections were carried out using Trans-IT 2020 transfection reagent (Mirus) following the manufacturer's instructions. Plasmids expressing IAV NP, PB1, PB2, or PA proteins derived from Cal/09 strain were provided by Dr. Adolfo García-Sastre (Icahn School of Medicine at Mount Sinai, NY, USA). C-terminal V5-tagged plasmid for β5 was procured from DNASU Plasmid Repository (The Biodesign Institute/Arizona State University, Tempe, AZ, USA). β5 Human siRNA Oligo Duplex was bought from Origene (Origene Technologies, Inc., Rockville, MD, USA). Proteasome inhibitors MG132 (30uM, Sigma-Aldrich) and Bortezomib (1uM, Thermo Fisher Scientific), protein synthesis inhibitor Cycloheximide (50ug/ml, Thermo Fisher Scientific) were procured commercially.

NP mutant plasmids

Two regions of the NP body domain were mutated: NP-Mutant 1 (E46A, K48A) and NP-Mutant 2 (D101A) (14) by nested PCR with mutations introduced in the primers to amplify. Briefly, for the desired mutations of specific amino acids, PCR amplification with different oligonucleotides was used for nested PCR of the NP gene. Individual clones containing the mutations were confirmed by sequencing the entire PCR-amplified DNA to ensure that additional mutations were not introduced (15). Next, 293T cells were transfected with the wild-type or mutant NP plasmids.

Retroviral silencing

Retroviral silencing vectors encoding BIK shRNA and the corresponding control vector were procured from Origene (Origene Technologies, Inc.). The efficacy of the shRNA in suppressing BIK expression was validated in HAECs. Amplification and purification of plasmid DNA, as well as packaging of the retroviral particles, were conducted in Phoenix cells following the manufacturer's instructions. Briefly, Phoenix packaging cells were transfected with the retroviral constructs, and supernatants containing packaged virus particles were harvested between 48 to 96 hours post-transfection. The supernatants were then centrifuged at 2000 rpm for 10 minutes to eliminate packaging cells, and aliquots of the viral supernatants were stored at -80°C until further use. HAECs were infected with the viral supernatant in

the presence of 10 μg/ml polybrene (Sigma-Aldrich). Stable cell lines expressing BIK and control shRNAs were established by selecting infected cells with 1 μg/ml puromycin (Calbiochem Inc., San Diego, CA, USA).

Mini-Genome assay

The mini-genome assay was conducted by transfecting HEK293T cells with 10, 100 or 200 ng of NP plasmid, 50ng each of PB1, PB2, and PA of PR/8 (pDZ or pCAGGS backbone), pPol-I reverse complement firefly Luc ORF (100 ng), and SV40 Renilla (25 ng) reporter plasmids using TransIT-2020 transfection reagent (Mirus). At 24 hours post-transfection, cells were lysed using 1x lysis buffer (Promega, Madison, WI, USA), and luciferase activity in the supernatant was measured employing the Dual Luciferase Assay Kit (Promega). Firefly luciferase values were normalized for transfection efficiency to renilla luciferase values. The data are expressed as firefly luciferase activity relative to WT-NP, presented as mean ± standard error mean (SEM). Results are representative of a minimum of three independent experiments.

RNA isolation and quantitative RT-PCR

Total RNA extraction from HAECs/NHBEs at designated time points was carried out using the RNeasy Mini kit (Qiagen, Hilden, Germany) following the manufacturer's recommended protocol. Briefly, cultured cell pellets were homogenized in Buffer RLT and centrifuged at 12,000 rpm for 3 minutes. The supernatant was mixed with an equal volume of 70% ethanol and loaded onto a RNeasy mini column. The column was washed sequentially with buffer RW1 and buffer RPE before RNA elution using nuclease-free water. The purity of the isolated RNA was assessed using a NanoDrop 8000 spectrophotometer and software v2.3.2 (Thermo Fisher Scientific). Subsequently, TaqMan-based quantitative real-time polymerase chain reaction (qRT-PCR) analysis was performed on QuantStudio 3 Real-Time PCR System (Applied Biosystems, CA, USA) utilizing the TaqMan™ RNA-to-C_T™ 1-Step Kit (Applied Biosystems) in accordance with the manufacturer's instructions. β-Actin or GAPDH were employed as internal controls. The difference in cycle threshold (C_t) values was calculated using the 2^-ΔΔCt method to determine the relative fold change in the expression levels of the candidate genes.

Expression data is presented as the average from three independent experiments. PCR reaction conditions included cDNA synthesis at 48°C for 15 minutes, enzyme activation at 95°C for 10 minutes followed by amplification of cDNA for 40 cycles with TaqMan (95°C for 15 seconds, 60°C for 1 minute). The primers are listed in supplemental Table 1 and procured from Applied Biosystems.

Western blot analysis and immunoprecipitation

Protein lysates for western blot were prepared by lysing cellular pellets in radioimmunoprecipitation assay (RIPA) buffer (Boston BioProducts, Inc., Milford, MA, USA) supplemented with protease inhibitor cocktail (Sigma Aldrich). Lung homogenates were centrifuged at 12,000 g for 15 minutes at 4°C. Lysates were used to determine total protein content using the colorimetric BCA assay (Pierce BCA protein assay kit, Thermo Fisher Scientific). 50µg protein/lane was electrophoresed on SDS-PAGE gel, blotted to PVDF membrane, blots were blocked with 5% non-fat dried milk dissolved in tris-buffered saline with Tween 20 (TBST) buffer at RT for 1 hour, followed by overnight incubation with appropriately diluted primary antibodies at 4°C (antibody details provided in Supplemental Table 2). After thorough washing with TBST, the blots were hybridized with horseradish peroxidase-conjugated secondary antibody at RT for 1 hour and detected using enhanced chemiluminescence (SuperSignal™ West Pico PLUS Chemiluminescent Substrate, Thermo Fisher Scientific). Protein bands were visualized using chemiluminescence imager (iBright1500, Invitrogen, Carlsbad, CA, USA). β-actin or GAPDH were used to normalize protein expression and densitometric analysis of band intensities was performed using ImageJ v1.53t software (NIH). For coimmunoprecipitation (coIP), protein lysates were immunoprecipated using protein A agarose beads (Cell Signaling Technology, Inc., Danvers, MA, USA) conjugated to specific antibody. Cell lysates were incubated with antibody overnight at 4 °C followed by 1 hour incubation with protein A agarose beads at 4 °C. Subsequently, the immunoprecipitates were eluted and subjected to Western blot analysis. The details of the primary and secondary antibodies used are provided in supplemental Tables 2 and 3.

Ubiquitin immunoprecipitation

Treated HEK293T cells were washed with PBS followed by collection in PBS, centrifuge at 4°C at 2,500 RPM for 1 minute. The pellet was resuspended in 60µl of lysis buffer consisting of 2% SDS in tris-buffered saline (TBS) with 1µl ubiquitin aldehyde and 1µl deubiquitinating enzyme inhibitor per sample. This was

followed by sonication and addition of 600µl 1X TBS to each sample. Samples were boiled for 10 minutes at 100°C followed by BCA and immunoprecipitation. Briefly, specific antibody was added to the protein homogenate and placed on rotator overnight at 4°C. Added 40µl protein A agarose beads and rotated at 4°C for at least two hours, centrifuged 1 minute at 2,500 RPM at 4°C. Removed supernatant and added 700µl PBS and repeated wash 3 times. Added 40µl 2X dye/loading buffer to the pellet and heated at 95°C for 5 minutes and used for SDS-PAGE and WB.

Immunofluorescence

For immunostaining, cells grown on Falcon® 4-well Culture Slide (Corning, NY, USA) were subject to specific treatments followed by fixation using 4% paraformaldehyde. The cells were blocked with 3% BSA containing 0.2% Triton X-100 for 1 h at RT, incubated overnight at 4°C with specific primary antibody followed by fluorochrome-tagged secondary antibody conjugated for 1 h at RT. The cells were then mounted with DAPI-containing mounting media (Abcam, Cambridge, UK) for nuclear staining. Immunofluorescence was imaged using (Olympus IX81, Olympus Corporation), with a with the SlideBook 6.0 acquisition software. Mouse lung sections were deparaffinized followed by blocking, and then incubated with appropriately diluted primary antibody overnight and with secondary antibody for 1 hour at RT. After washing in PBS, sections were mounted in DAPI-containing mounting media (Abcam), viewed using Olympus IX81 microscope (Olympus Corporation), and images were analyzed using SlideBook 6.0 digital microscopy software. The details of the antibodies used are provided in supplemental Table 2.

SNP genotyping

Genotyping was performed for rs738276 in DNA samples isolated from NHBEs from 50 individuals and PBMCs from 105 individuals using standard TaqMan genotyping thermal cycling protocol on QuantStudio 3 (Applied Biosystems). The Genotype clustering data was analyzed using the Taqman Genotyper software (Applied Biosystems).

Proteomics analysis

HEK293T cells were grown to 80% confluency and infected with vehicle or 0.1 MOI IAV. Cell lysates were immunoprecipitated using anti-BIK antibody. Immunoprecipitates were electrophoresed on SDS-PAGE gel and protein bands were excised and submitted to Taplin Biological Mass Spectrometry Facility (Harvard Medical School, Boston, USA) for proteomic analysis. Three independent experiments were conducted. Proteomics data were analyzed for FDR and Log2 values using R-software (CCIC Mass Spectrometry and Proteomics Facility, The Ohio State University, Columbus, USA). Identified peptides were selected based on their highest representation and signal intensity. The Volcano plot was performed using GraphPad Prism v10.0.2 software (GraphPad Software Inc., La Jolla, CA, USA) to identify the top BIK-interacting proteins increased or inhibited by IAV infection.

Statistical analysis

Statistical analyses were performed using either GraphPad Prism 10 (GraphPad Software, Inc.) or SAS (SAS 9.4, Cary, NC, USA). Grouped results from at least three different sets of experiments were expressed as mean with standard error of means (SEM), and differences between groups were assessed for significance by Student's t test when the data were available in only two groups. When the data were available in more than two groups, analysis of variance (ANOVA) was used to perform pair-wise comparison. When significant main effects were detected ($p < 0.05$), Fisher least significant difference test was used to determine the differences between groups. The *in vitro* and mouse data are expressed as the mean group value ± SEM and analyzed using GraphPad Prism v10.0.2 (GraphPad Software Inc.). Data from each group of treatment were subjected to two-tailed unpaired student's *t* test. The criterion for significance was $p < 0.05$ in all studies. For the *in vivo* studies, differences in body weight were expressed as mean group value ± SEM. Percentage survival of mice was analyzed using the Log-rank (Mantel-Cox) test.

Data, Materials, and Software Availability

All study data are included in the article and/or Supplemental Information.

Supplementary Table 1: Assay IDs (Thermo Fisher Scientific, Waltham, MA, USA) of the used TaqMan probes used for qRT-PCR analysis.

**Gene**	**Assay ID**
β-Actin (Mouse)	Mm02619580_g1
BIK (Human)	Hs00609635_m1
BIK (Mouse)	Mm00476123_m1
Bad (Human)	Hs00188930_m1
Bak (Human)	Hs00832876_g1
Bcl-2 (Human)	Hs04986394_s1
Bcl-xl (Human)	Hs00236329_m1
GAPDH (Human)	Hs99999905_m1

Supplementary Table 2: Details of the primary antibodies used for protein expression analysis.

Antibody	Manufacturer	Cat. No.	Host	Application	Dilution
α-tubulin	Invitrogen, Carlsbad, CA, USA	322588	Mouse	IF	1:200
β5	Abcam, Cambridge, UK	ab3330	Rabbit	IF<br>WB	1:100<br>1:3000
β-actin	Merck & Co., Inc., Rahway, NJ, USA	A5441	Mouse	WB	1:5000
Bad	Cell Signaling Technology, Danvers, MA, USA	9268	Rabbit	WB	1:1000
Bak	Abcam, Cambridge, UK	ab104124	Mouse	WB	1:1000
Bax	Cell Signaling Technology, Danvers, MA, USA	2772	Rabbit	WB	1:1000
Bcl-2	Abcam, Cambridge, UK	ab182858	Rabbit	WB	1:1000
BIK	Invitrogen, Carlsbad, CA, USA	PA5-78873	Rabbit	IF<br>WB	1:100<br>1:500
Bim	Stressgen Biotechnologies, CA, USA	AAP-330	Rabbit	WB	1:500
Flag	Abcam, Cambridge, UK	ab205606	Rabbit	WB	1:1000
GAPDH	Cell Signaling Technology, Danvers, MA, USA	5174	Rabbit	WB	1:2000
GFP	Invitrogen, Carlsbad, CA, USA	MA5-15256	Mouse	IF	1:200
HA-Tag	Cell Signaling Technology, Danvers, MA, USA	2367	Mouse	WB	1:1000
HA-Tag	Cell Signaling Technology, Danvers, MA, USA	3724	Rabbit	WB	1:1000
IRF-1	Cell Signaling Technology, Danvers, MA, USA	8478	Rabbit	WB	1:1000
K48Ub	Cell Signaling Technology, Danvers, MA, USA	8081	Rabbit	WB	1:1000
Lamin A/C	Cell Signaling Technology, Danvers, MA, USA	4777	Mouse	WB	1:1000
M2	GeneTex, Irvine, CA, USA	GTX125951	Rabbit	WB	1:1000
Noxa	Invitrogen, Carlsbad, CA, USA	MA1-41000	Mouse	WB	1:1000
NP	Novus Biologicals, Centennial, CO, USA	NBP2-16965	Rabbit	IF<br>WB	1:200<br>1:2000
NS1	Abcam, Cambridge, UK	ab101278	Rabbit	WB	1:1000
PA	GeneTex, Irvine, CA, USA	GTX125933	Rabbit	WB	1:1000
PB1	GeneTex, Irvine, CA, USA	GTX125923	Rabbit	WB	1:1000
PB2	GeneTex, Irvine, CA, USA	GTX125926	Rabbit	WB	1:1000
Ub	Cell Signaling Technology, Danvers, MA, USA	2367	Mouse	WB	1:1000
V5-Probe	Santa Cruz Biotechnology, Dallas, TX, USA	sc-271944	Mouse	WB	1:500

Supplementary Table 3: Details of the secondary antibodies used for protein expression analysis.

Antibody	Manufacturer	Cat. No.	Host	Application	Dilution
Anti-Mouse IgG-HRP	Jackson ImmunoResearch Laboratories, Inc., West Grove, PA, USA	115-035-003	Goat	WB	1:5000
Anti-Rabbit IgG-HRP	Jackson ImmunoResearch Laboratories, Inc., West Grove, PA, USA	111-035-003	Goat	WB	1:5000
Anti-rabbit IgG-HRP (Conformation Specific)	Cell Signaling Technology, Danvers, MA, USA	5127	Mouse	WB	1:2000
Anti-Mouse IgG (H+L), Alexa Fluor 488	Invitrogen, Carlsbad, CA, USA	A-11001	Goat	IF	1:200
Anti-Mouse IgG (H+L), Alexa Fluor 546	Invitrogen, Carlsbad, CA, USA	A-11018	Goat	IF	1:200
Anti-Rabbit IgG (H+L), Alexa Fluor 488	Invitrogen, Carlsbad, CA, USA	A-11070	Goat	IF	1:200
Anti-Rabbit IgG (H+L), Alexa Fluor 546	Invitrogen, Carlsbad, CA, USA	A-11071	Goat	IF	1:200

Supplemental Figure 1.

A

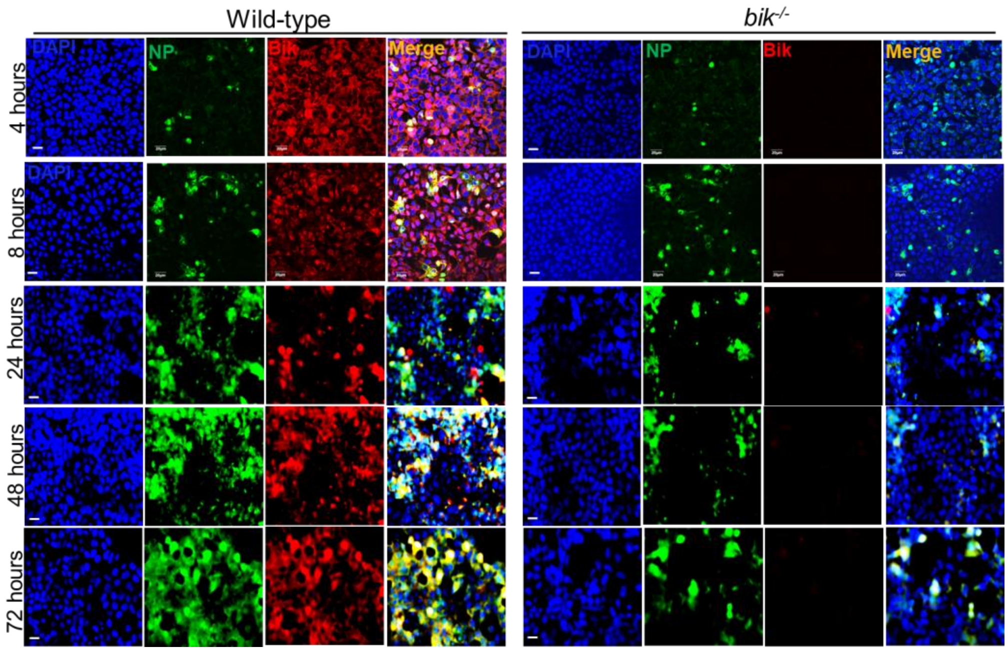

B

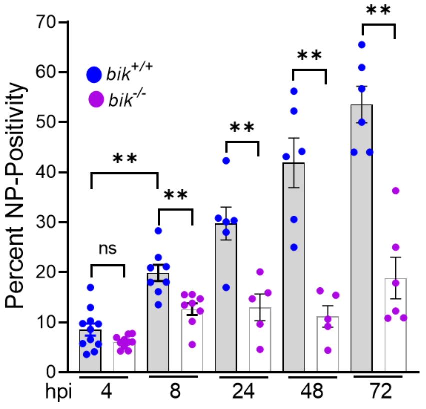

C

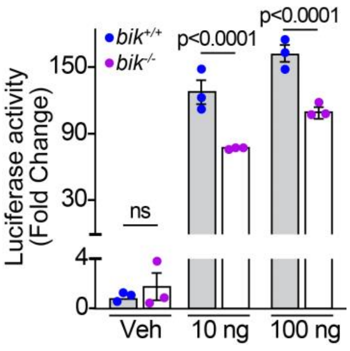

(A) Primary mouse AECs were infected with PR/8 at 0.1 MOI. Cells were immunostained for influenza A nucleoprotein (NP) and imaged right after individual experimental time points. Scale bar=20µm. **(B)** Percent NP-positivity at 4-72 hpi using ImageJ software. n=6-11/group. * $p < 0.05$, ** $p < 0.01$, Two-tailed t-test. **(C)** Parent and CRISPR/Cas9 *BIK* knockout HEK293T cells were co-transfected with using PR/8 PA, PB1, PB2, and NP plasmids on a pDZ backbone, and firefly and renilla luciferase plasmids. The luciferase activity indicating polymerase activity was measured using Promega kit. n=3. Two-way ANOVA. Error bars: Mean SEM.

Supplementary Figure 2.

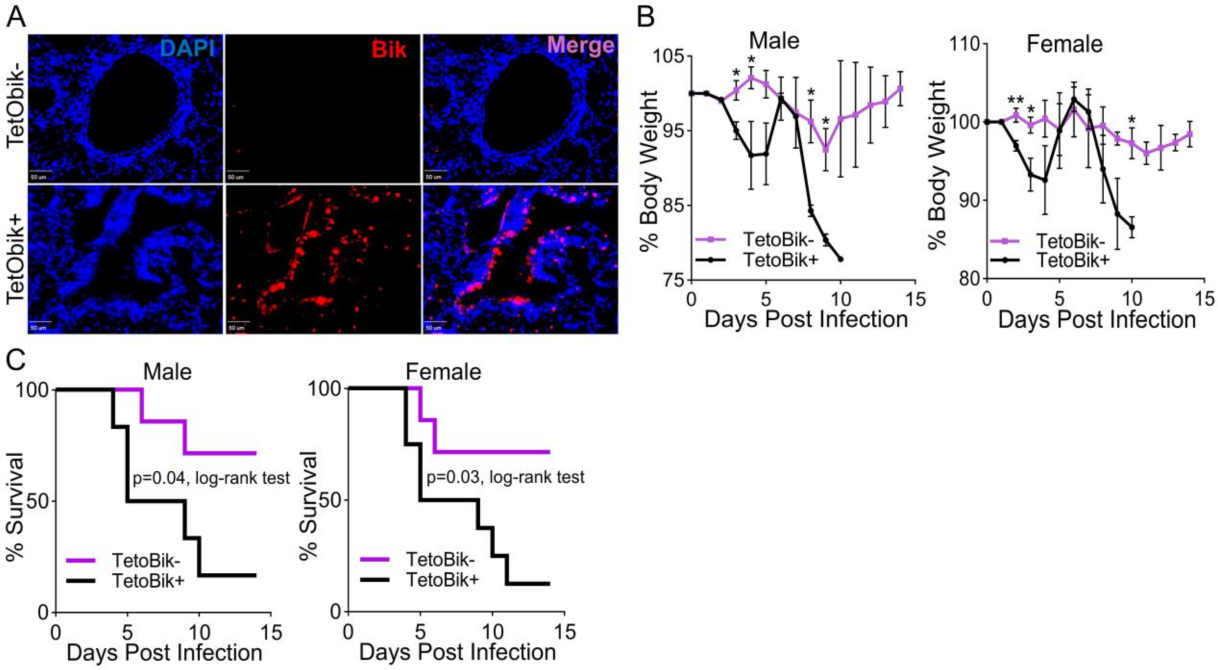

(A) CCSP-IndBIK and CCSP transgenic mice in *BIK* knockout (*bik*$^{-/-}$) background were kept with 400 mg/l doxycycline containing water for 5 days. The lung tissues were immunostained using anti-BIK antibody and analyzed for BIK protein expression using fluorescent microscopy. n=4. Eight weeks old male and female CCSP-IndBik transgenic mice in *bik*$^{-/-}$ background and their littermates were infected 250 pfu PR/8 intranasally and kept on 400 mg/l doxycycline containing water *ad libitum*. Mice were monitored for **(B)** changes in body weight and **(C)** survival (Log-rank (Mantel-Cox) test) for a period of 14 days. n=5 males and 5 to 8 females per group. t-test; Mean ± SEM; * $p < 0.05$, ** $p < 0.01$.

Supplemental Figure 3.

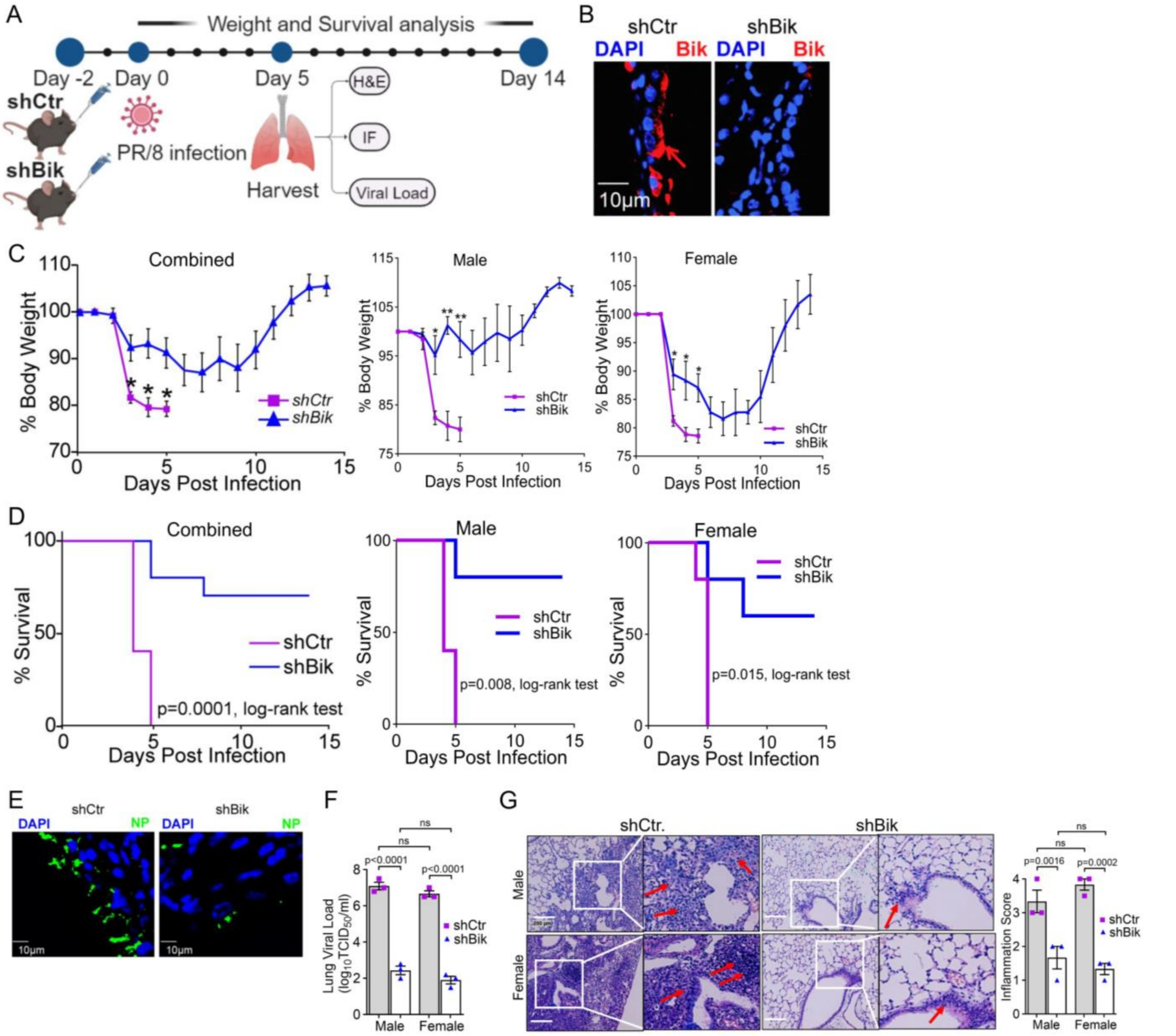

Depleting BIK in the airways mitigates IAV-induced morbidity and mortality in mice. **(A)** C57BL/6 mice were treated with shControl or shBIKik on day -2 and infected with 250 pfu PR/8 on day 0. Figure made with Biorender.com. **(B)** Lung tissues were immunostained using an anti-BIK antibody on 1 dpi and analyzed by fluorescent microscopy. Red fluorescence = BIK. (**C**) Mean body weights were compared using Two-tailed t-test; n=5/group for male and female; and **(D)** % survival was analyzed using Log-rank test. n=5/group for male and female. On 5 dpi, lung tissues were **(E)** immunostained for viral NP expression and analyzed by confocal microscopy, green fluorescence = NP in the airways, and **(F)** lung

homogenates were used to analyze viral loads using the median tissue culture infectious dose (TCID50). Two-way ANOVA; n=3 per group. **(G)** H&E-stained lung tissues were analyzed for lung inflammation and inflammation score on 5 dpi. Two-way ANOVA; n=3 per group. Red arrows indicate infiltrates of inflammatory cells. Error bars: Mean ± SEM.

Supplemental Figure 4.

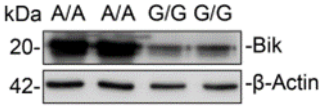

Peripheral blood mononuclear cells (PBMCs) were grown on culture plates, genotyped for the *BIK* SNP, and protein lysates analyzed for BIK level by Western blot. n=2.

Supplemental Figure 5

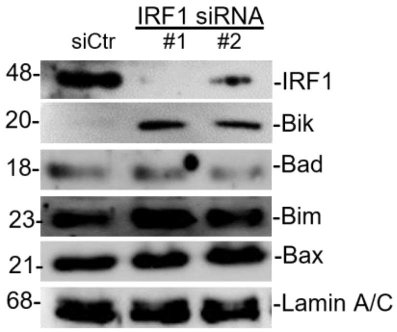

Silencing IRF1 in $NHBE_{G/G}$ increased BIK expression enhancing viral replication and inflammation. NHBEs with the GG variant of the *BIK* SNP ($NHBE_{G/G}$) were transfected with siControl or siIRF1 followed by infection with 0.1 MOI Cal/09. Protein lysates were analyzed for BIK, Bad, Bim, Bax, and IRF1 levels using Western blot.

Supplemental Figure 6.

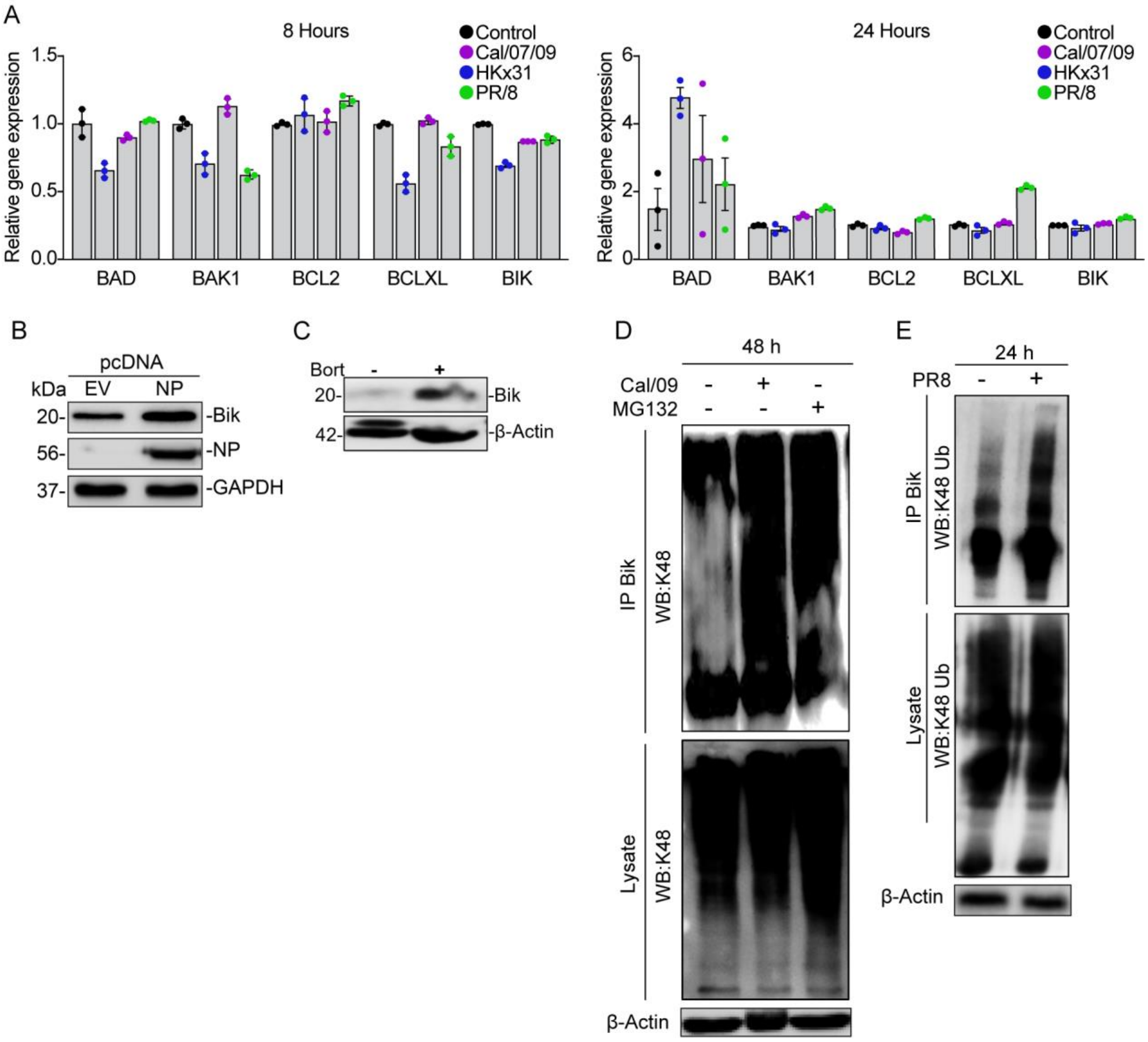

(A) Human AECs were infected with 1 MOI of the indicated IAVs, and mRNA was analyzed using qRT-PCR 8 and 24 h later. **(B)** A549 cells were transfected with 1.0µg empty vector (EV) or NP expressing plasmids. Protein lysates were analyzed for the expression of BIK and NP levels by Western blot. **(C)** HEK293T cells were treated with vehicle or 50µg/ml bortezomib for 6 h and cell lysates were analyzed for BIK protein levels by Western blot. **(D)** HEK293T cells were infected with vehicle or 1 MOI Cal/09 for 48 h or 20µM MG132 for 6 h. BIK immunoprecipitates were immunoblotted for K48 ubiquitin protein by Western blot. **(E)** HEK293T cells were infected with 1 MOI PR/8 and BIK immunoprecipitates were immunoblotted for K48 ubiquitin by Western blot.

Supplemental Figure 7.

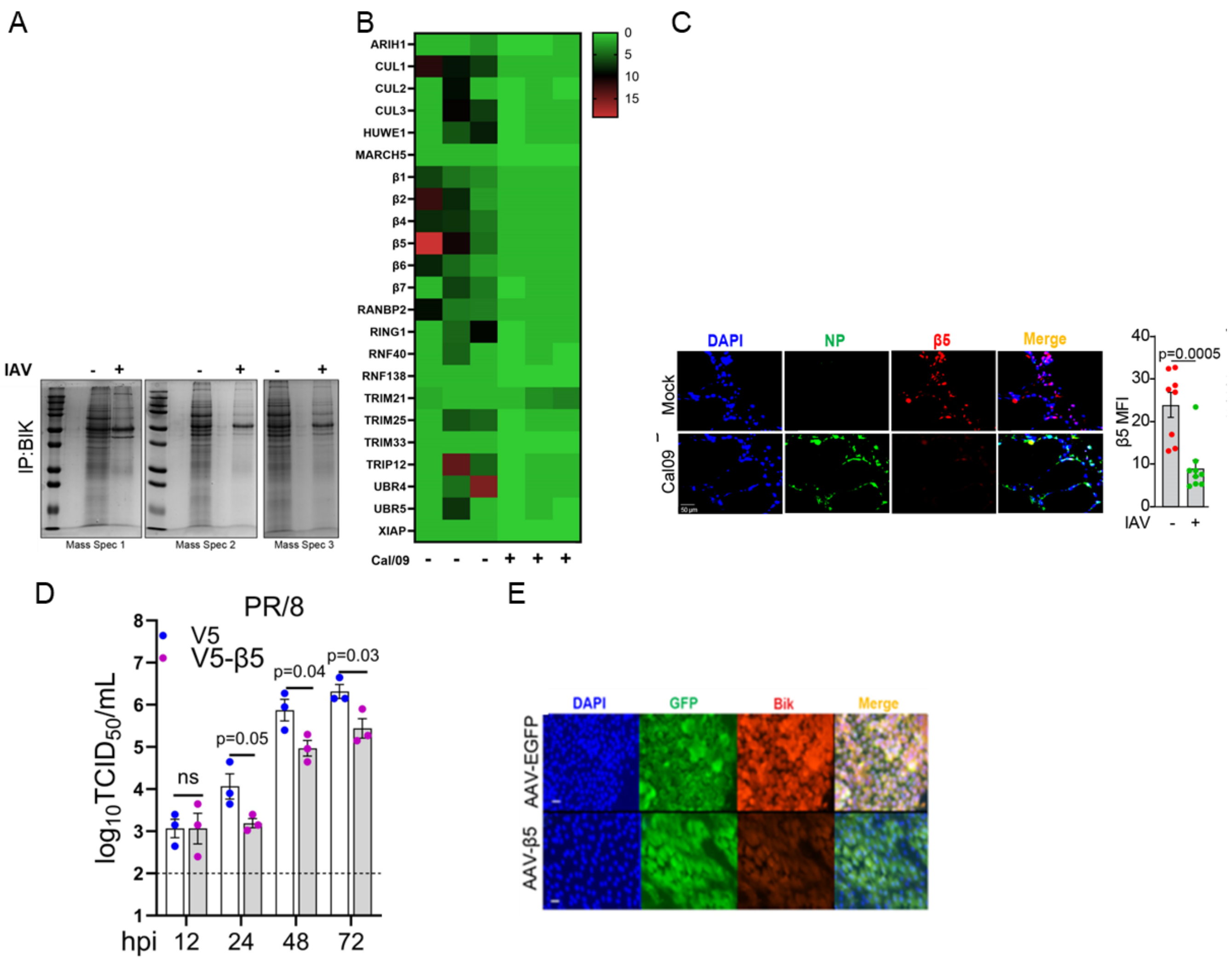

(A) HEK293T cells were infected with mock or 0.1 MOI Cal/09. Forty-eight hours later, cell lysates were immunoprecipitated with anti-BIK antibody and electrophoresed on SDS-PAGE gels. Protein bands were cut and submitted for proteomic analysis. Identified peptides were selected based on their highest representation. **(B)** The proteomics data was used to create a heat-map for the top BIK-interacting proteasomal proteins inhibited by influenza A virus infection. An equal amount of BIK was pulled from vehicle- or Cal/09-infected cells and submitted for proteomics analysis. β5 shows the highest inhibition by influenza A infection. **(C)** Human precision-cut lung slices (hPCLS) were infected with mock (PBS) or Cal/09. Forty-eight hours later paraffin embedded hPCLS slices were immunostained for β5 and NP and analyzed using fluorescent microscopy. The mean fluorescence intensity (MFI) for β5 was analyzed using ImageJ software. n=8 per group. **(D)** A549 cells were transfected with V5 or V5-β5 followed by infection

with 0.1 MOI PR/8. Viral titers were compared using $TCID_{50}$ at the indicated time points. N=3. **(E)** ALI-differentiated $NHBE_{A/A}$ cultures were infected with 0.1 MOI Cal/09 and treated with 10^11 genome copies/well of AAV-GFP or AAV-β5. Cultures were immunostained at 72 hpi. Scale bar = 20μm.

Supplemental Figure 8

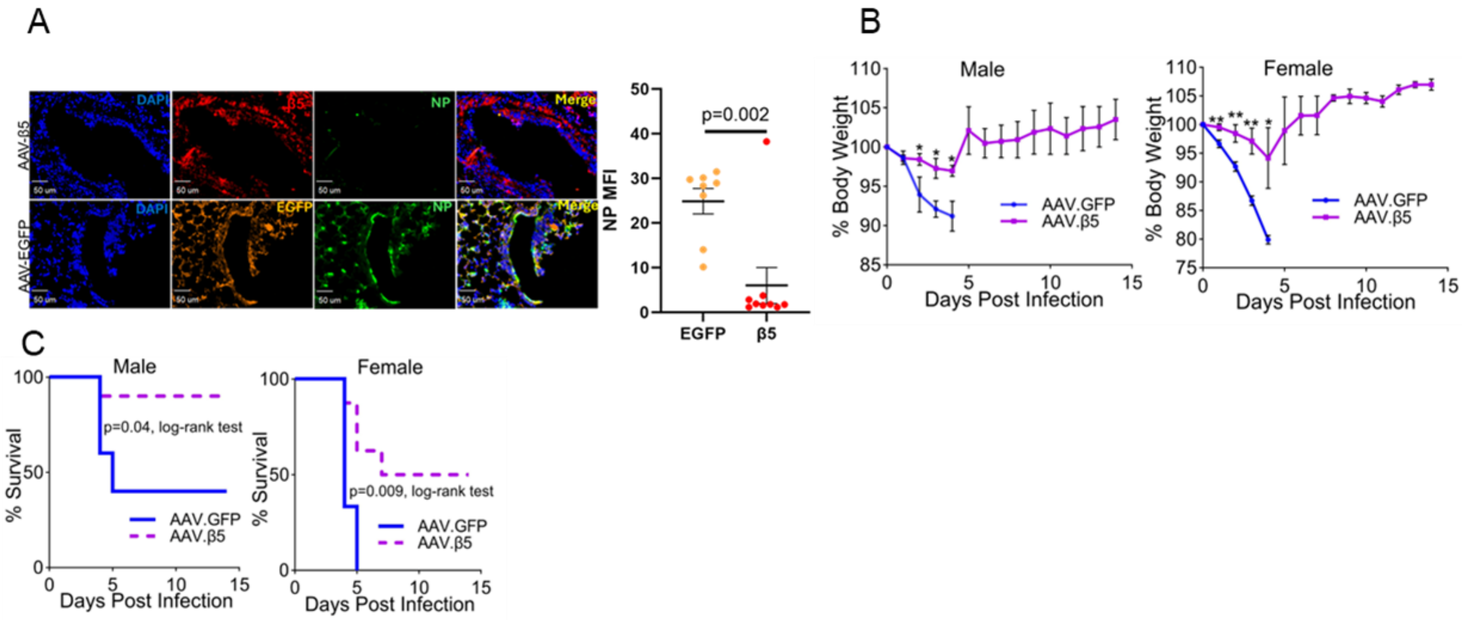

Wild-type C57BL/6 mice were treated with 10^11 genome copies of AAV-GFP or AAV-β5 intranasally for 5 days. Mice were infected with a lethal dose of PR/8 (250 pfu). **(A)** On day 5 post PR/8 infection paraffin-embedded lung tissues were immunostained for EGFP, β5, and NP expression and analyzed using fluorescent microscopy. Mean fluorescent intensity was compared using ImageJ software. n=8-9/group. Two-tailed t-test. Graphs were made using GraphPad Prism. **(B)** Mice were weighed daily and **(C)** monitored for survival over a period of 14 days after infection. * $p<0.05$, ** $p<0.01$. t-test; n=4-9 per group. Error bars: Mean ± SEM.

Supplemental Figure 9.

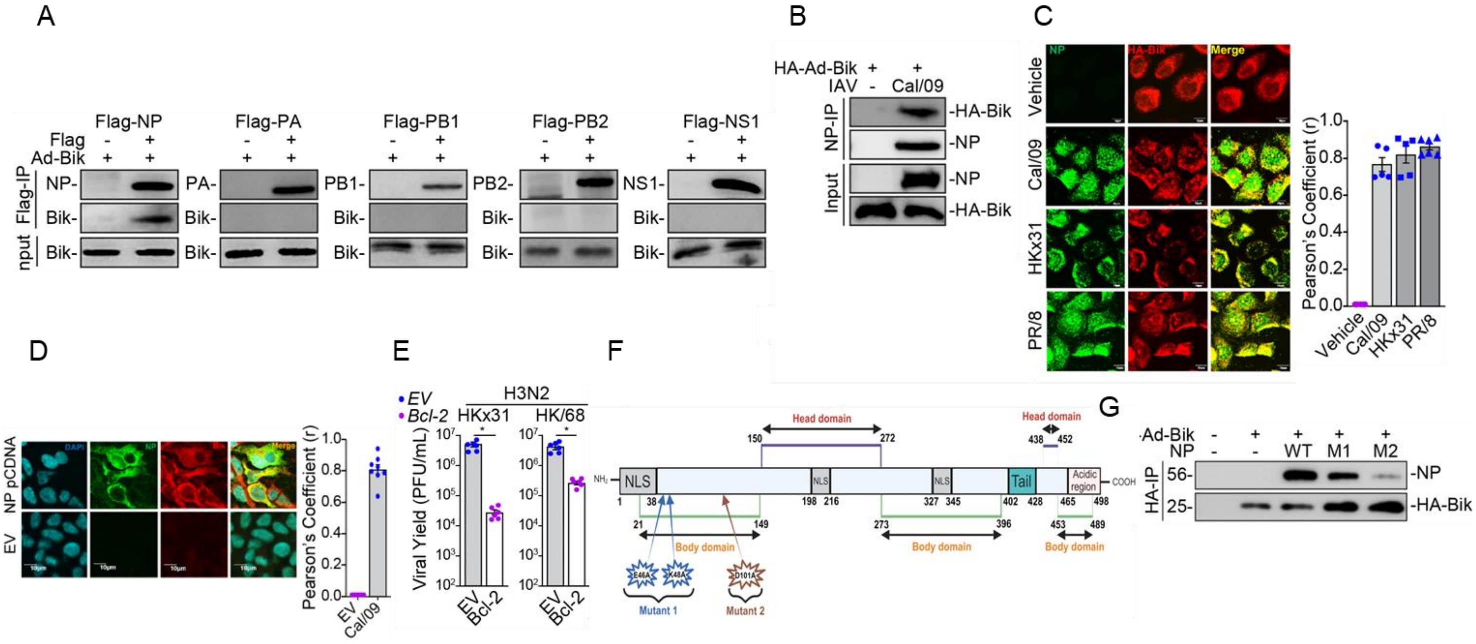

(A) HEK293T cells were transfected with empty vector (EV) or the indicated Flag-tagged IAV protein constructs. Cell lysates were treated with RNase A and immunoprecipitated with anti-Flag antibody. The immunoprecipitates were analyzed for BIK and indicated viral proteins expression by Western blot. **(B)** Human AECs were infected with vehicle or 0.1 MOI Cal/09. Twenty-four hours later cells were infected with 50 MOI Ad-BIK. Protein lysates were immunoprecipitated with anti-NP antibody and analyzed for the indicated proteins by Western blot. **(C)** HAECs were infected with 50 MOI Ad-BIK and infected with vehicle or 0.1 MOI of the indicated IAV strains. Cells were immunostained for BIK and NP and analyzed for BIK/NP co-localization using confocal microscopy 24 hpi. Pearson's coefficient values were analyzed using ImageJ. n=6. **(D)** HEK293T cells were transfected with empty vectors or plasmids expressing Cal/09 NP. Cells were immunostained for BIK and NP and analyzed for BIK/NP co-localization using confocal microscopy. Pearson's Coefficient analysis for BIK/NP colocalization is shown on the right-hand side. n=8 per group. **(E)** HEK293T cells transfected with EV or Bcl-2 expression vector and infected with 0.1 MOI of the indicated IAV strains. Virus titers were compared in the apical washes using plaque assay at 72 hpi. **(F)** Map of IAV NP showing NP motifs that were mutated in the body domain. **(G)** HEK293T cells were transfected with 1μg of EV or WT or mutant NP plasmid constructs. Twenty-four hour later,

cells were infected with 50 MOI HA-Ad-BIK. Protein lysates were immunoprecipitated with anti-HA antibody and analyzed for NP and HA-BIK protein levels by Western blot.

Supplemental Figure 10

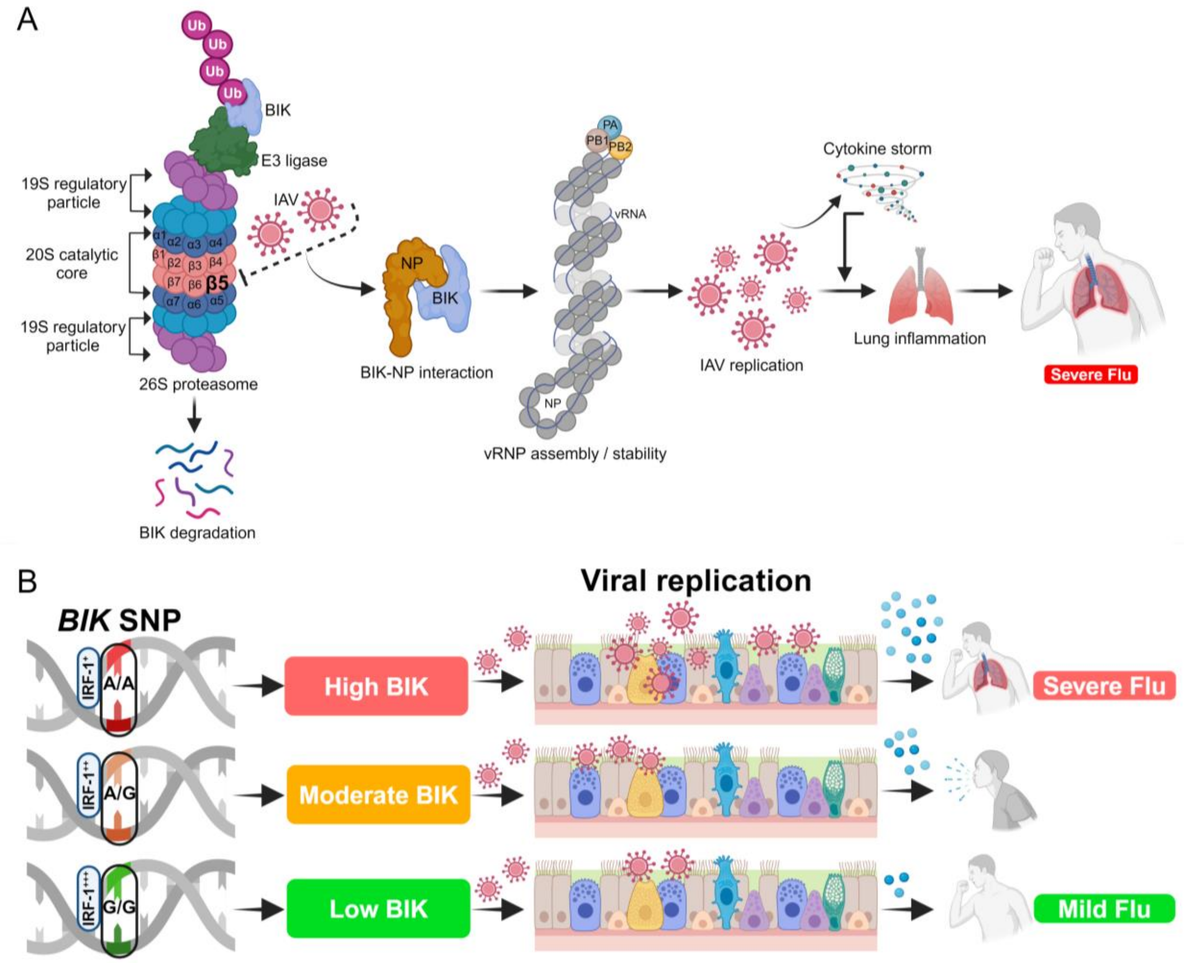

Proposed model. **(A)** IAV stabilizes BIK in AECs by blocking the catalytic subunit of the 20S proteasome, β5, to inhibit the degradation of ubiquitin-conjugated BIK. IAV-stabilized BIK interacts with and stabilizes viral NP, allowing NP to assemble viral ribonucleoprotein (vRNP) components for efficient viral replication. **(B)** The *BIK* rs738276 SNP regulates BIK expression. This SNP, influenced by differential IRF-1 binding (39), alters BIK expression. Individuals with the AA genotype show elevated BIK levels and exhibit enhanced viral replication in the AECs. This, in turn, contributes to elevated pro-inflammatory cytokine levels and greater influenza disease severity. Figure made with Biorender.com.

List of Abbreviations:

A549: Human lung epithelial cells

Ad-BIK: Adenoviral expression vector for BIK

AECs: Airway epithelial cells

ALI: Air-liquid interface

ANOVA: Analysis of variance

ATCC: American type culture collection

$bik^{-/-}$: bik-knockout

BIK: Bcl-2-interacting killer

Cal/09: A/California/04/2009 (IAV H1N1)

CMV: Cytomegalovirus

Co-IP: Co-immunoprecipitation

C_t: Cycle threshold

DMEM: Dulbecco's Modified Eagle Medium

dox: Doxycycline.

dpi: Days post infection

ER: Endoplasmic reticulum

EV: Empty vector

FBS: Fetal bovine serum

H&E: Hematoxylin and eosin

HAECs: Human airway epithelial cells

HEK293T: Human embryonic kidney cells

HK/68: A/Hong Kong/1/1968 (IAV H3N2)

HKx31: A/Hong Kong/x31 (IAV H3N2)

hPCLS: Human precision-cut lung slices

hpi: Hours post infection

IACUC: Institutional animal care and use committee

IAVs: Influenza A viruses

IP: Immunoprecipitation

IRF-1: Interferon regulatory factor-1

MAECs: Mouse airway epithelial cells

MDCK: Madin Darby canine kidney cells

MFI: Mean fluorescence intensity

NHBEs: Normal human bronchial epithelial cells

$noxa^{-/-}$: noxa-knockout

NP: Nucleoprotein

PBMCs: Peripheral blood mononuclear cells

PBS: Phosphate-buffered saline

PCR: Polymerase chain reaction

pfu: Plaque-forming unit

PR/8: A/Puerto Rico/8/1934 (IAV H1N1)

qRT-PCR: Quantitative real-time polymerase chain reaction

RIPA: Radioimmunoprecipitation assay

RT: Room temperature

SEM: Standard error mean

SNP: Single nucleotide polymorphism

TBS: Tris-buffered saline

TBST: Tris-buffered saline with Tween 20

$TCID_{50}$: Median tissue culture infectious dose

UPS: Ubiquitin proteasome system

vRNP: Viral ribonucleoprotein

WT: Wild type

References

1. E. K. Allen *et al.*, SNP-mediated disruption of CTCF binding at the IFITM3 promoter is associated with risk of severe influenza in humans. *Nat Med* **23**, 975-983 (2017).
2. T. King, A. Mejias, O. Ramilo, M. E. Peeples, The larger attachment glycoprotein of respiratory syncytial virus produced in primary human bronchial epithelial cultures reduces infectivity for cell lines. *PLoS Pathog* **17**, e1009469 (2021).
3. M. L. Fulcher, S. Gabriel, K. A. Burns, J. R. Yankaskas, S. H. Randell, Well-differentiated human airway epithelial cell cultures. *Methods Mol Med* **107**, 183-206 (2005).
4. Z. O. Shi, M. J. Fischer, G. T. De Sanctis, M. R. Schuyler, Y. Tesfaigzi, IFN-gamma, but not Fas, mediates reduction of allergen-induced mucous cell metaplasia by inducing apoptosis. *J Immunol* **168**, 4764-4771 (2002).
5. T. Cruz, A. L. Mora, M. Rojas, Determination of Senescent Myofibroblasts in Precision-Cut Lung Slices. *Methods Mol Biol* **2299**, 139-145 (2021).
6. Y. Bai *et al.*, Cryopreserved Human Precision-Cut Lung Slices as a Bioassay for Live Tissue Banking. A Viability Study of Bronchodilation with Bitter-Taste Receptor Agonists. *Am J Respir Cell Mol Biol* **54**, 656-663 (2016).
7. J. E. McLean, E. Datan, D. Matassov, Z. F. Zakeri, Lack of Bax prevents influenza A virus-induced apoptosis and causes diminished viral replication. *J Virol* **83**, 8233-8246 (2009).
8. Y. A. Mebratu *et al.*, Bik Mediates Caspase-Dependent Cleavage of Viral Proteins to Promote Influenza A Virus Infection. *Am J Respir Cell Mol Biol* **54**, 664-673 (2016).
9. M. T. Ling *et al.*, Mannose-binding lectin contributes to deleterious inflammatory response in pandemic H1N1 and avian H9N2 infection. *J Infect Dis* **205**, 44-53 (2012).
10. M. H. Reed LJ, A simple method of estimating fifty-percent endpoints. *Am J Hyg* **27**, 493–497 (1938).
11. L. Wang *et al.*, Interplay between MDM2, MDMX, Pirh2 and COP1: the negative regulators of p53. *Mol Biol Rep* **38**, 229-236 (2011).

12. J. M. van den Brand *et al.*, Severity of pneumonia due to new H1N1 influenza virus in ferrets is intermediate between that due to seasonal H1N1 virus and highly pathogenic avian influenza H5N1 virus. *J Infect Dis* **201**, 993-999 (2010).
13. M. Germain, J. P. Mathai, G. C. Shore, BH-3-only BIK functions at the endoplasmic reticulum to stimulate cytochrome c release from mitochondria. *J Biol Chem* **277**, 18053-18060 (2002).
14. A. M. Davis, J. Ramirez, L. L. Newcomb, Identification of influenza A nucleoprotein body domain residues essential for viral RNA expression expose antiviral target. *Virology journal* **14**, 22 (2017).
15. S. K. Biswas, P. L. Boutz, D. P. Nayak, Influenza virus nucleoprotein interacts with influenza virus polymerase proteins. *J Virol* **72**, 5493-5501 (1998).
